# Nerve-associated Schwann cell precursors contribute extracutaneous melanocytes to the heart, inner ear, supraorbital locations and brain meninges

**DOI:** 10.1007/s00018-021-03885-9

**Published:** 2021-07-18

**Authors:** Marketa Kaucka, Bara Szarowska, Michaela Kavkova, Maria Eleni Kastriti, Polina Kameneva, Inga Schmidt, Lucie Peskova, Alberto Joven Araus, Andras Simon, Jozef Kaiser, Igor Adameyko

**Affiliations:** 1 Max Planck Institute for Evolutionary Biology, Plön, Germany; 2 Department of Neuroimmunology, Center for Brain Research, Medical University Vienna, Vienna, Austria; 3 Central European Institute of Technology BUT, Brno, Czech Republic; 4 Department of Physiology and Pharmacology, Karolinska Institutet, Stockholm, Sweden; 5 Department of Histology and Embryology, Masaryk University, Brno, Czech Republic; 6 Department of Cell and Molecular Biology, Karolinska Institutet, Stockholm, Sweden

**Keywords:** Extracutaneous pigment cell, Targeted recruitment, Endothelin 3 and endothelin receptor B, Glial precursor, Peripheral nerves, Hypopigmentation-associated deafness

## Abstract

Melanocytes are pigmented cells residing mostly in the skin and hair follicles of vertebrates, where they contribute to colouration and protection against UV-B radiation. However, the spectrum of their functions reaches far beyond that. For instance, these pigment-producing cells are found inside the inner ear, where they contribute to the hearing function, and in the heart, where they are involved in the electrical conductivity and support the stiffness of cardiac valves. The embryonic origin of such extracutaneous melanocytes is not clear. We took advantage of lineage-tracing experiments combined with 3D visualizations and gene knockout strategies to address this long-standing question. We revealed that Schwann cell precursors are recruited from the local innervation during embryonic development and give rise to extracutaneous melanocytes in the heart, brain meninges, inner ear, and other locations. In embryos with a knockout of the *EdnrB* receptor, a condition imitating Waardenburg syndrome, we observed only nerve-associated melanoblasts, which failed to detach from the nerves and to enter the inner ear. Finally, we looked into the evolutionary aspects of extracutaneous melanocytes and found that pigment cells are associated mainly with nerves and blood vessels in amphibians and fish. This new knowledge of the nerve-dependent origin of extracutaneous pigment cells might be directly relevant to the formation of extracutaneous melanoma in humans.

**Supplementary Information:**

The online version contains supplementary material available at 10.1007/s00018-021-03885-9.

## Introduction

Melanocytes inhabit the skin, hair follicles, and the iris of the eye [1–4]. These cells are responsible for the protection from UV radiation damage and provide colour to the skin or fur, which is crucial for various aspects of the animal’s life, including display and camouflage [4–7]. However, melanocytes are also present in locations deep inside the body that are not directly exposed to UV radiation. These extracutaneous pigment cells are found in the *stria vascularis* of the inner ear, in the leptomeninges, *substantia nigra* and *locus coeruleus* of the brain [8, 9]. Additionally, melanocytes reside in the cardiac valves, in the septum and in the major arteries and veins of the heart [10, 11].

The presence and the density of melanocytes in the cardiac valves correlate with the stiffness and mechanical properties of the valves, which suggests that melanocytes support their proper function [12]. Furthermore, cardiac melanocytes control the amount of atrial reactive oxygen species, which play a role in the electrical and structural remodelling of the heart [13]. Indeed, Levin and co-authors demonstrated that melanocytes of the pulmonary veins and the heart are atrial arrhythmia triggers [14]. Despite the emerging knowledge about the function of cardiac melanocytes, this is still an emerging field. More experiments are yet to be performed to reveal the entire spectrum of the roles, which cardiac and other extracutaneous melanocytes play in the developing embryo and during adulthood.

Among many functions, melanocytes have a crucial role during the development and function of *stria vascularis* in the inner ear [9, 15]. Mice and humans lacking melanocytes inside the inner ear suffer from congenital hearing loss, likely due to the impaired development or collapse of the *stria vascularis*. This causes the inability to produce the endocochlear potential [15, 16]. Numerous disorders exhibiting deafness or arrhythmia in combination with hypopigmentation further highlight the importance of the presence of melanocytes, for instance, Waardenburg syndrome (specifically type 4, also known as Waardenburg–Shah syndrome), Alezzandrini syndrome, Vogt–Koyanagi–Harada disease, Tietz syndrome and ABCD syndrome (albinism, black lock of hair, cell migration disorder of the neurocytes of the gut, and sensorineural deafness syndrome) [14, 17–22] and others (for a comprehensive overview, see [23]). Interestingly, the proper function of the inner ear does not depend on the production of the pigment by melanocytes as the albino mice and albino guinea pigs do have mostly unaffected hearing. Nevertheless, the pigment seems to have a protective function during age-related or noise-induced hearing loss [24–28].

Finally, melanocytes are also known to be phagocytic, cytokine-producing and antigen-presenting cells resembling the morphological and functional properties of dendritic cells, thus being accounted among the first-line innate immunity response [29–31].

Recently, the embryonic origin of cutaneous melanocytes has been readdressed, which revealed that these pigment cells arise both from the neural crest cells (NCCs) migrating to the skin from the dorsal neural tube and from the multipotent Schwann cell precursors (SCPs) residing on the surface of developing nerve fibres [32–34]. Furthermore, this SCP-dependent origin of pigment cells is evolutionarily conserved among fish, birds, and mammals [32–36]. Still, the origin of extracutaneous melanocytes was not investigated in relation to innervation and associated SCPs.

The core molecular players associated with the melanocyte fate include PAX3, SOX10, MITF, SNAI2, EDNRB, EDN3, KIT and KITL [37–43]. Briefly, the neural crest cells (NCCs) express transcription factor *Pax3* before their delamination from the neural tube and in the absence of *Pax3*, multiple NCC-related defects are observed [44]. The melanocyte specification is further driven by the cross-regulatory interactions of the transcription factors MITF and SOX2 [45]. MITF is considered a master regulator of the establishment of melanocyte fate but cannot induce full differentiation without the presence of SOX10 [46]. Only together, SOX10 and MITF induce the expression of *Dct* and *Tyrosinase*. The ligand EDN3 and its counterpart endothelin receptor B (EDNRB) are reported to regulate the expansion of melanocyte progenitors and affect their differentiation into mature melanocytes [47].

Melanocytes can emerge in different locations from migratory neural crest cells or SCPs. This dual cellular origin complicates our understanding of melanocyte biology. The development of the neural crest cells occurs during a short time window at the end of the neurulation, and melanocytes that are born directly from the neural crest might not be sufficient in numbers to populate all locations of a fast-growing embryo [32, 45, 48–50]. The possibility to recruit melanocytes from the innervation at the later stages of development provides a more sustainable source of the cellular material and might provide the opportunity to deliver melanocytes to the locations that are not accessible via the neural crest or melanoblast migration. Thus, the origin of such melanocytes can be connected to their local specialization and several non-canonical functions. Next, the capacity of SCPs to transform into melanocytes can result in a number of malignancies, including extracutaneous melanomas [51] and melanocytosis [52]. Therefore, knowing the origin of various populations of extracutaneous and even cutaneous melanocytes is essential for our understanding of specific disorders and for envisioning future treatment strategies.

In this study, we addressed the origin of extracutaneous melanocytes located in the heart, inner ear, brain meninges and other extracutaneous locations. We revealed that peripheral nerve-associated SCPs are an essential source of extracutaneous melanocytes in all investigated locations, and the disruption of EDNRB/EDN3 signaling results in the accumulation of nerve-associated melanoblasts not capable of leaving the nerves as well as dispersing into internal and external body locations. This provides the first understanding of the molecular machinery and developmental context of melanocyte appearance in deep body locations. Importantly, our findings lay the foundation for further investigation of the molecular mechanisms of targeted cell recruitment to develop new promising therapeutic strategies in hypopigmentation-associated deafness specifically related to mutations of *Ednrb* and *Edn3* genes. Furthermore, we investigated the evolutionary conservation of extracutaneous melanocyte association with blood vessels and nerves in the inner organs across analyzed species.

## Results

**Fig. 1:**
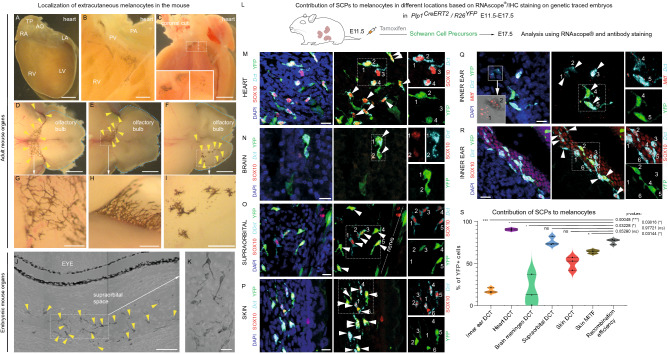
Extracutaneous melanocytes in the inner ear, the heart and the brain are recruited from Schwann cell precursors. **A**–**I** Extracutaneous melanocytes found in the heart (**A**–**C**) and the brain (**D**–**I**) of adult mice. **A** Overview of the heart that has been dissected in (**B**, **C**). Note that the melanocytes are found in the valves and also lining the walls of arteries and veins of the heart. **D**–**F** Melanocytes in the leptomeninges of the brain in the adult mouse are localized to the area of olfactory bulb (yellow arrowheads point at the pigmented areas or individual melanocytes). **G**–**I** Insets are magnifying the pigmented areas from (**D**–**F**). **J**, **K** Pigmented melanocytes reside in the supraorbital space of the mouse embryonic head at E17.5, proximally to the eyeball. Yellow arrowheads point at the individual melanocytes in (**J**). White dotted rectangle in (**J**) shows the area magnified in (**K**), 90˚ rotated. *LV* left ventricle, *LA* left atrium, *RV* right ventricle, *RA* right atrium, *AO* aorta, *PV* pulmonary valve, *PA* pulmonary artery, *DRG* dorsal root ganglion. **L** This scheme represents the design of the lineage-tracing experiment using *Plp1*^*CreERT2*^*/R26*^*YFP*^ mice. Tamoxifen injection was administered at E11.5, which triggered recombination and the expression of the permanent fluorescent marker (YFP; visualized as a green signal) in Schwann cell precursors. The lineage tracing in *Plp1*^*CreERT2*^*/R26*^*YFP*^ was visualized by co-staining with anti-GFP antibody (**M**–**R**). The embryos were analyzed at E17.5 using RNAscope^®^ technology and immunohistochemistry (**M**–**R**), and quantified (**S**). The following locations were analyzed for the presence of *Dct*^+^ (and *Mitf*^+^) melanocytes: heart (**M**), brain (**N**), supraorbital region (**O**), skin (**P**), and the inner ear (**Q**, **R**). White arrowheads point at the individual melanocytes in (**M**–**R**), whereas the white dotted line shows the area magnified on the right side of each panel. For better orientation, the magnified melanocytes are numbered to distinguish the presence or absence of the YFP signal. The quantified differences in the contribution of SCPs to the extracutaneous melanocytes are visualized as a violin plot (**S**). The contribution of SCPs to melanocytes in different locations was calculated as a proportion of YFP^+^/*Dct*^+^ melanocytes from all *Dct*^+^ melanocytes. Recombination efficiency has been assessed as a proportion of YFP^+^/SOX10^+^ cells from all SOX10^+^ cells of the SC lineage within the nerves. For each location, three embryos (*n* = 3) from the independent litters were analyzed. Mean ± SEM; *p*-value for each location are: inner ear *Dct* (17.47 ± 1.784; 0.00048), heart *Dct* (90.11 ± 0.8285; 0.03016), brain meninges *Dct* (16.51 ± 10.88; 0.03228), supraorbital *Dct* (76.02 ± 3.041; 0.97721), skin *Dct* (50.47 ± 4.511; 0.05280), skin *Mitf* (63.78 ± 1.277; 0.03144), and the recombination efficiency in peripheral nerves (75.87 ± 1.822). Scale bars are 1 mm (**A**, **C**, **D**–**F**), 250 µm (**B**), 50 µm (**G**–**J**), 10 µm (**K**–**R**)

Recently, it became evident that melanocytes inhabit multiple extracutaneous locations, including the heart and inner ear. We performed additional analysis in adult mice and found pigmentation in brain meninges, dorsal root ganglia (DRGs), spinal cord and supraorbital spaces between the eyeballs and the brain (Fig. 1A–K and Supplementary Figure 1). Some locations were pigmented only sporadically and locally (DRGs, spinal cord) (Supplementary Figure 1), whereas the other places always showed the presence of stable pigmentation (inner ear, heart, brain meninges, supraorbital) (Fig. 1A–K).

**Fig. 2:**
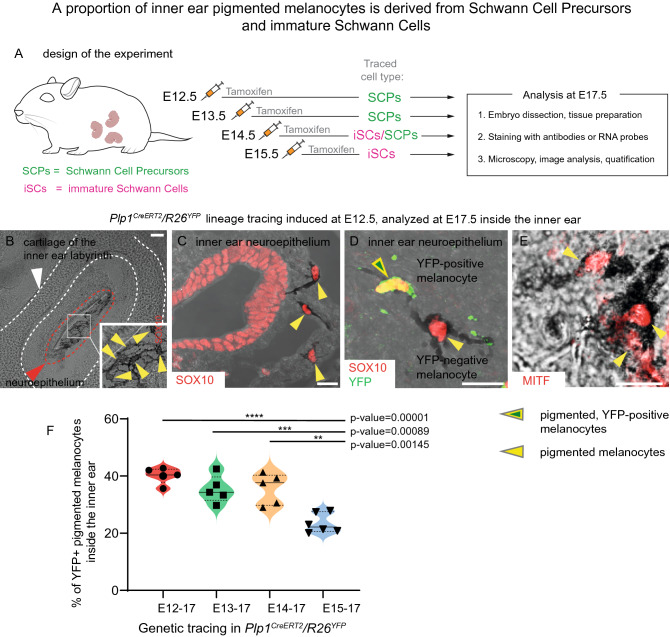
A proportion of the inner ear melanocytes is derived from Schwann cell precursors and immature Schwann cells. **A** The scheme represents the design of the lineage-tracing experiment using *Plp1*^*CreERT2*^*/R26*^*YFP*^ mice. Tamoxifen injections were administered either at E12.5, E13.5, E14.5 or E15.5, which triggered recombination and the permanent expression of fluorescent marker (YFP) in either Schwann cell precursors (marked with green “SCPs”) or immature Schwann cells (marked with magenta “iSCs”). Note that immature Schwann cells start to form around E15.5 differentiating from SCPs. However, their exact proportions and capacity to generate other cell types stay undetermined due to different opinions in the scientific community regarding the markers identifying these cells clearly. All obtained embryos were analyzed at E17.5 using immunohistochemistry (**B**–**E**), and the contribution of the SCP lineage to melanocytes was quantified and visualized in (**F**). **B**–**E** Pigmented melanocytes were identified using transmitted light based on the presence of pigmentation and typical cell morphology inside of the inner ear, proximally to the neuroepithelium. **C**–**E** Melanocytes co-express expected markers *Sox10* and *Mitf*, and the lineage tracing in *Plp1*^*CreERT2*^*/R26*^*YFP*^ was visualized by co-staining with the anti-GFP antibody (**D**). **F** Contribution of Schwann Cell Precursors and immature Schwann cells to melanocytes of the inner ear was assessed by performing the analysis of genetic tracing in E17.5 embryos using *Plp1*^*CreERT2*^*/R26*^*YFP*^ mouse line. For every time-point, a minimum of five different embryos from three independent litters were evaluated. White square in (**B**) shows the area magnified in the inset, and the yellow arrowheads point at the individual pigmented melanocytes. White arrowhead and white dotted lines mark the cartilage of the inner ear in (**B**). Red arrowhead and the red dotted line mark neuroepithelium of the inner ear in (**B**). Yellow arrowheads point at individual pigmented melanocytes (in **C**–**E**) expressing one of the typical melanocytes markers: SOX10 in (**C**, **D**) or MITF in (**E**). Yellow-green arrowhead points at pigmented YFP^+^ melanocyte in (**D**). Mean ± SEM and *n* for each stage are: E12.5 (40.14 ± 1.237, *n* = 5), E13.5 (35.34 ± 2.115, *n* = 5), E14.5 (35.56 ± 2.438, *n* = 5) and E15.5 (23.42 ± 1.392, *n* = 6). Scale bars are 20 µm (**B**) and 10 µm (**C**, **D**)

To understand the origin of melanocytes in such locations with stable pigmentation pattern, we employed genetic lineage tracing using the *Plp1*^*CreERT2*^*/R26*^*YFP*^ mouse strain (Figs. 1L–S, 2). In this strain, recombination is activated specifically in Schwann cell precursors if the tamoxifen is administered after the neural crest cells cease their migration between E9.5 and E10.5 [32, 53–56]. The specificity of *Plp1*^*CreERT2*^*/R26*^*YFP*^ has been extensively addressed in our previous publications [32, 45] and also verified by the experiments performed by other labs, for instance, in comparison with *Tyr*^*CreERT2*^, which labeled a different pool of melanocytes if injected at the same time as in case of *Plp1*^*CreERT2*^ [57]. We induced genetic recombination in SCPs at post-neural crest migration time-points to address the contribution of nerve-associated SCPs to the extracutaneous melanocyte pools in the developing mouse embryo (Figs. 1L–S, 2).

We utilized the embryos heterozygous both for the *CreERT2* recombinase allele and *R26YFP* reporter allele to avoid any possible tracing artefacts related to the leakage of recombination before administration of tamoxifen (we specifically controlled for that). The melanocyte fate was detected by RNAscope^®^ and immunohistochemistry for well-established markers of melanocyte fate SOX10, *Dct* and *Mitf* (Fig. 1M–R), and by the presence of pigment-transporting melanosomes (Fig. 2B–E).

The analysis of genetic recombination in the mouse embryonic nerves at E17.5 showed that 75.87% ± 3.15 SEM of all SOX10^+^ peripheral glial cells were traced when tamoxifen was injected at E11.5 (Fig. 1S). The analysis of the proportion of traced *Dct*^+^ cells at E17.5 in different embryonic locations (brain meninges, heart, inner ear, skin, supraorbital) showed a varying contribution of nerve-associated SCPs, ranging from relatively moderate (inner ear) to extraordinarily high (heart) (Fig. 1L–S, Supplementary Information 1). The investigated *Dct*^+^ cells also showed the expression of *Mitf* and *Sox10*, which confirmed their melanocyte fate (Fig. 1M–R). Not all *Dct*^+^*/*SOX10^+^ cells contained pigment granules at the time of the analysis at E17.5 developmental stage, especially in the inner ear and the heart (Supplementary Figure 2). Overall, these results show that most of the investigated extracutaneous locations receive melanocytes from both cellular sources: migratory neural crest and nerve-associated SCPs. The high tracing proportion observed in cardiac *Dct*^+^*/*SOX10^+^ cells (see Fig. 1S) might indicate that SCPs represent their predominant origin with a minimal possible contribution from the migratory neural crest.

Next, we focused on analyzing the dynamics of the developmental origin of the fully pigmented melanocytes in the mouse embryonic inner ear (Fig. 2). When genetic lineage tracing was induced at E12.5 in SCPs, we observed 40.14 ± 1.24% (mean ± SEM) of YFP^+^ melanocytes and 35.34 ± 2.12% if injected at E13.5. Injection at E14.5 gave us 35.56 ± 2.44% of traced pigmented cells and appeared reduced to 23.42 ± 1.39% of YFP^+^ melanocytes when the pregnant mice were injected at E15.5 (Fig. 2F). Thus, the level of SCP recruitment into melanocytes appeared reduced at E15.5, suggesting that the recruitment of glial cells from the nerve into the melanocyte fate occurs predominantly during a specific developmental time window. The proportion of traced pigmented inner ear melanocytes observed in this experiment appeared higher as compared to the proportion of all traced *Dct*^+^ cells (pigmented + unpigmented) when the tamoxifen was induced at E11.5 (reflected in Fig. 1S). We suggest that pigmented and unpigmented populations might have a different balance of origins in the inner ear, with variations in their spatial distribution and corresponding source, which altogether fits the recent data published by Bonnamour et al. [58].

**Fig. 3:**
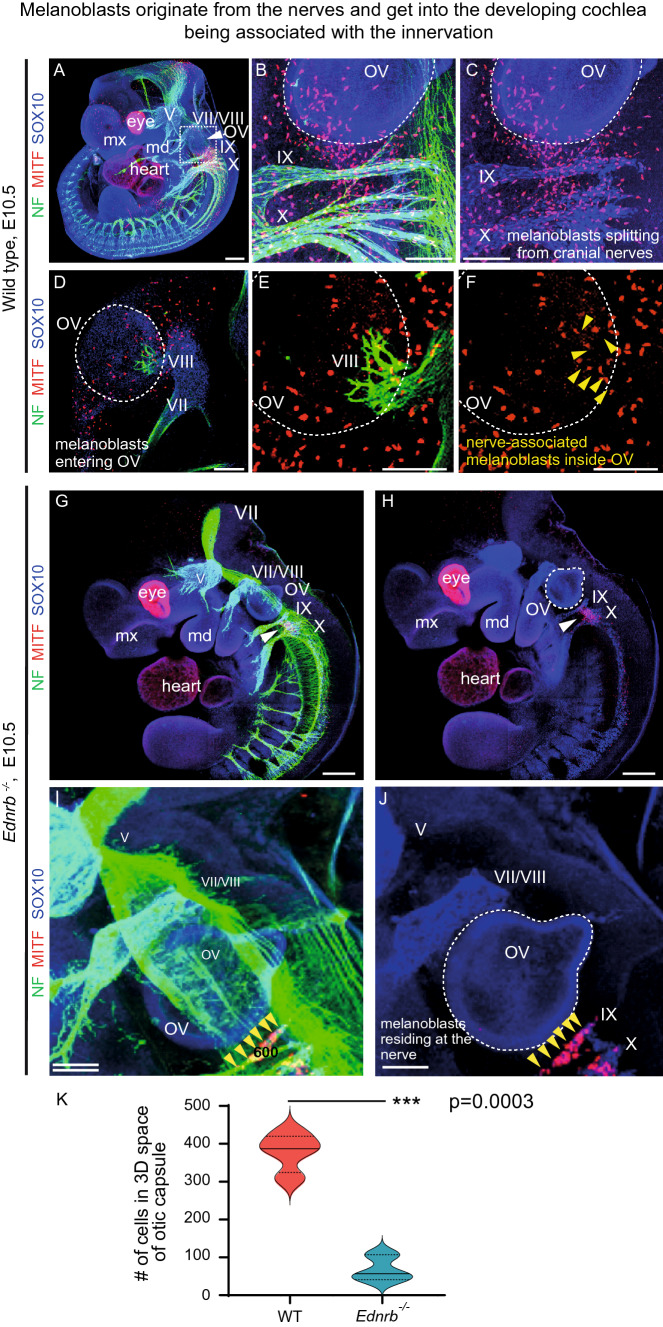
Melanoblasts originate from the nerves and get into the developing cochlear being associated with the innervation. **A**–**F** E10.5 control mouse embryo and **G**–**J** E10.5 *Ednrb*^*−/−*^ mouse embryo. **A** The 3D visualization of E10.5 embryo with stained innervation (green, NF and blue, SOX10) and melanoblasts (red, MITF). Roman numbers mark corresponding cranial nerves. White dotted square shows the area magnified in (**B**, **C**). **B**, **C** The area of the developing otic vesicle (outlined with white dotted line) contain entering melanoblasts, which originate and split from the local cranial nerves. **D**–**F** The otic vesicle with the entering cranial nerve VIII. **E**, **F** Magnified area of the otic vesicle from (**D**) shows entering cranial nerve VIII together with nerve-associated melanoblasts and melanoblasts that split from the nerve. **G**, **H** A 3D rendering of an *Ednrb* mutant embryo shows significantly decreased amount of melanoblasts (red, MITF) emerging from the cranial nerves (green, NF and blue, Sox10). **I**, **J** In the mutant embryo, the magnified area with the developing otic vesicle shows the absence of melanoblasts in the forming inner ear despite the presence of developing nerve fibers. Note that low numbers of melanoblasts are born within the cranial nerves IX and X. Nevertheless, the remaining melanoblasts do not split from the innervation. Technical note: In addition to being expressed by melanoblasts, *Mitf* is expressed by developing cardiac muscle and retinal pigmented epithelium. Yellow arrowheads point at the individual MITF^+^ melanocytes in (**F**, **I**, **J**). In total, three mutant and three control embryos from three independent litters have been analyzed. *mx* maxilla, *md* mandible, *OV* otic vesicle. Scale bars are 250 µm (**A**, **G**, **H**), 100 µm (**B**–**F**, **I**, **J**). **K** Graph shows the differences in melanocytes present in the 3D-area of the otic region in *Ednrb*^*−/−*^ and WT control embryos. Each condition *n* = 3, *p* = 0.0003, mean ± SEM in WT (379.75 ± 25.37) and *Ednrb*^*−/−*^ (68.33 ± 19.87)

To understand how and when the nerve-derived melanoblasts reach the inner ear during the early formation of the otic vesicle, we performed whole-mount immunostaining of several E10.5 mouse embryos and visualized the developing peripheral nervous system with associated SCPs and MITF^+^ cells (Fig. 3). Consistent with our previous findings [45], we observed that melanocytes appeared within the developing nerve fibres of the facio-acoustic ganglion complex (cranial nerves and ganglia VII–VIII) and cranial nerves IX and X proximally to the otic vesicle (Fig. 3A–F). Some melanoblasts entered the developing otic vesicle (future inner ear) together with the VIII cranial nerve (the vestibulocochlear nerve, also called auditory vestibular nerve) that mediates the connection from the inner ear to the brain. The VIII cranial nerve enters the otic vesicle at around embryonic day 10.5, which is also the first time when we detected the presence of melanoblasts in the ear based on the expression of the transcription factor MITF.

To challenge the system and to impair the recruitment of the melanoblasts from the nerves to the otic vesicle, we utilized the *endothelin receptor type B (Ednrb)* knockout strategy (Fig. 3G–J) [59, 60]. This knockout mouse strain resembles the phenotype of human congenital disorders such as Hirschsprung’s disease (HSCR) and Waardenburg syndrome type IV. In human patients suffering from HSCR, sensorineural hearing loss is one of the major symptoms. We analyzed this mutant mouse strain at E10.5 with the help of 3D reconstruction of confocal stacks and detected a complete absence of melanoblasts entering the otic vesicle in three mutant embryos (Fig. 3G–J) in comparison to three E10.5 controls (Fig. 3A–F). Although the cranial nerves were developed and present, only a fraction of melanoblasts appeared in strict association with the IX and X cranial nerves. These melanoblasts did not migrate from the nerve into the developing inner ear. This finding might explain the cause of deafness in *Ednrb*-defined HSCR patients and the *Ednrb* knockout mice.

**Fig. 4:**
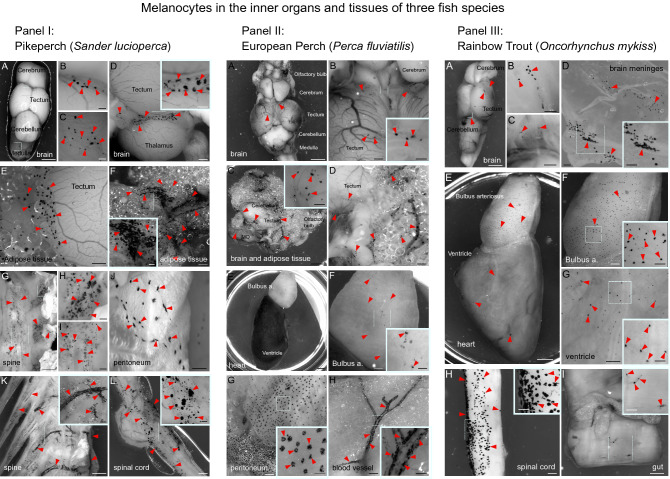
Pigmented melanocytes in the inner organs and tissues of three fish species. *Panel I* (left) shows representative pictures from the pikeperch (*Sander lucioperca*). *Panel II* (middle) shows the internal pigmentation in the european perch (*Perca fluviatilis*). *Panel III* (right) represents internal pigment distribution in the rainbow trout (*Oncorhynchus mykiss*). Pigmented melanocytes are found in different quantities on the brain surface, overlying meninges and surrounding adipose tissue, adjacent to the spine, in the peritoneum, gut and in the heart. Red arrowheads point at the pigmented areas or at the individual pigmented cells. *Panel I* (left; pikeperch): **A** brain, white dotted square is magnified in (**B, C**). **D** Brain, white dotted square is magnified in the inset. **E** Brain with the adjacent adipose tissue. **F** Adipose tissue associated with the brain; white dotted square marks the area magnified in the inset. **G** Spine, white dotted squares show areas magnified in (**H, I**). **J** Peritoneum. **K** Spine, white dotted square shows the area magnified in the inset. **L** Spinal cord is covered by the pigmented spinal meninx; white dotted square shows the area magnified in the inset. *Panel II* (middle, european perch): **A–D** Brain with the adjacent adipose tissue. **E, F** Different regions of the heart. **G** Peritoneum. **H** Branching blood vessel. (**B, D, F, G, H**) White dotted square shows areas magnified in the insets. *Panel III *(right; rainbow trout): **A** Brain, white dotted square is magnified in (**B, C**). **D** Pigmented brain meninx. **E–G** Heart, dotted white squares mark areas magnified in (**F, G**). **H** Spinal cord and **I** gut, white dotted squares mark areas magnified in the insets. Scale bars: pikeperch panels—1 mm (**A, G, K**), 500 µm (**D–F, I–J, L**, inset in **K**), 200 µm (**B, C, H**, insets in **D, F, L**); european perch panels—1 mm (**A, C, E**), 500 µm (**B, D, F–H**), 200 µm (insets in **B, C, F–H**); rainbow trout panels—1 mm (**A, E, I**), 500 µm (**F–H**), 200 µm (**B–D**, insets in **D, F–I**)

**Fig. 5:**
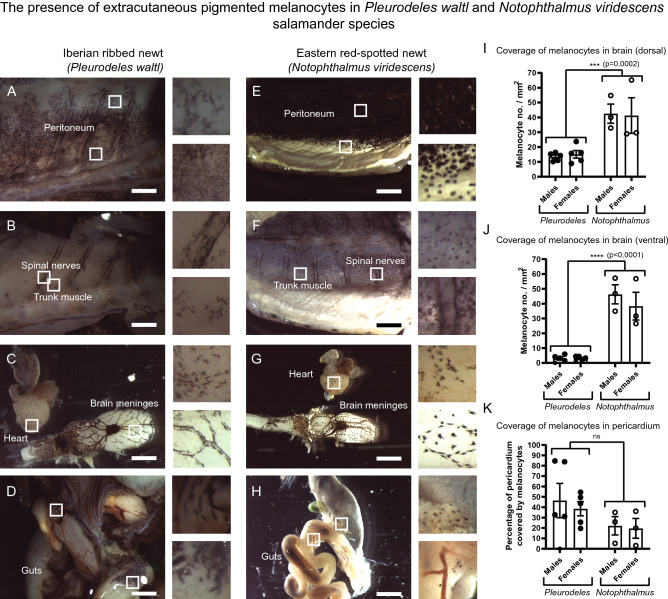
The presence of extracutaneous pigmented melanocytes in *Pleurodeles waltl* and *Notophthalmus viridescens* salamander species. **A**–**H** Extracutaneous melanocytes are consistently found in multiple inner structures of *Pleurodeles waltl* (**A**–**D**) and *Notophthalmus viridescens* (**E**–**H**). Pigmented melanocytes are found in the peritoneum (**A**, **E**), on the spinal nerves, trunk muscles (**B**, **F**), heart, brain meninges (**C**, **G**) and digestive system (**D**, **H**). **I**, **J** The melanocyte coverage of the brain meninges was significantly higher in *Notophthalmus viridescens* as compared to *Pleurodeles waltl* (two-way ANOVA dorsal view: *p* = 0.0002; ventral view, *p* < 0.0001). **I** Coverage of melanocytes in the dorsal brain meninges: mean ± SEM: male *Pleurodeles waltl* (13.41 ± 1.21), female *Pleurodeles waltl* (15.09 ± 2.57), female *Notophthalmus viridescens* (41.24 ± 11.99) and male *Notophthalmus viridescens* (42.54 ± 6.46). **J** Coverage of melanocytes in ventral brain meninges: mean ± SEM: male *Pleurodeles waltl* (2.975 ± 1.03), female *Pleurodeles waltl* (3.017 ± 0.67), female *Notophthalmus viridescens* (38.30 ± 9.35) and male *Notophthalmus viridescens* (46.29 ± 6.44). **K** The percentage of the pericardium area covered by melanocytes is greatly variable and does not differ significantly between sexes (two-way ANOVA *p* = 0.6943) or species (two-way ANOVA *p* = 0.1168). Mean ± SEM: male *Pleurodeles waltl* (46.45 ± 16.49), female *Pleurodeles waltl* (38.57 ± 6.79), female *Notophthalmus viridescens* (19.66 ± 9.55) and male *Notophthalmus viridescens* (22.09 ± 8.83). **I**–**K** Adult males and females of both *Notophthalmus viridescens* (*n* = 3 males, *n* = 3 females) and *Pleurodeles waltl* (*n* = 5 males, *n* = 5 females) were analyzed. Scale bars are 200 µm

Finally, to examine the evolutionary conservation of the extracutaneous melanocytes in the previously investigated locations, we performed an anatomical study of the inner organs and the nervous system in several fish and amphibian species, including pikeperch (*Sander lucioperca*) (Fig. 4—panel I), European perch (*Perca fluviatilis*) (Fig. 4—panel II), rainbow trout (*Oncorhynchus mykiss*) (Fig. 4—panel III), Iberian ribbed newt (*Pleurodeles waltl*) (Fig. 5A–D) and Eastern red-spotted newt (*Notophthalmus viridescens*) (Fig. 5E–H). We analyzed at least six individual animals per species and selected representative pictures. Consistently with the results in mice, we found extracutaneous melanocytes associated with multiple structures and tissues in all investigated species. Extracutaneous melanocytes were located in the peritoneum, immersed in the trunk muscle, scattered in the guts, in the heart and brain meninges (Figs. 4, 5A–H). We performed quantifications in two salamander species to explore inter-species and inter-individual variability (Fig. 5I–K). Pearson correlation of quantitative data on extracutaneous melanocytes, body length and weight of two salamander species indicated that inter-individual differences in melanocyte coverage of the brain and heart were not correlated with either the size, weight, or the sex of animals (data not shown). Inter-species comparisons showed that *Notophthalmus* have a higher melanocyte density in the brain than *Pleurodeles* (Fig. 5I, J). Intriguingly, melanocytes are more abundant in the dorsal part of the brain in *Pleurodeles* but equally distributed between the dorsal and ventral aspects of the brain in *Notophthalmus*. Analysis of the hearts showed scattered melanocytes in the aorta in both species (Fig. 5C, G), but melanocytes were observed in the atria of *Notophthalmus* only (Fig. 5G). The pericardium showed extensive variability in the number of melanocytes, which was not correlated to species or sex (Fig. 5L). This analysis suggests that the role of extracutaneous melanocytes might be related to the lifestyle and particular habitats of different amphibian species. In comparison with mice, extracutaneous melanocytes were more widely distributed and all locations were much stronger pigmented in salamanders (compare Figs. 1, 4 and 5. After a closer look, the specific distribution of melanocytes in all investigated extracutaneous locations in fish and amphibian species suggested that the pigment cells are largely associated with local blood vessels and nerves.

**Fig. 6:**
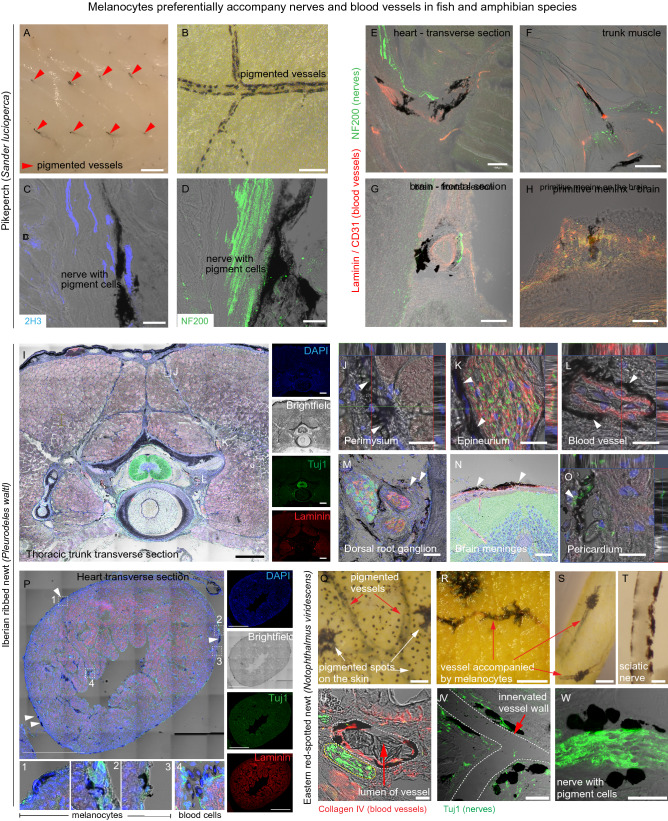
Melanocytes preferentially accompany nerves and blood vessels in fish and amphibian species. **A** Red arrowheads point at the pigmented neuro-muscular bundles within the muscles of the pikeperch (*Sander lucioperca*). **B** Melanocytes are lining the branching blood vessels in the pikeperch. **C**, **D** Immunohistological staining shows association of melanocytes with nerve fibers in the pikeperch. **E**–**H** Immunohistological staining shows association of extracutaneous melanocytes with the blood vessels (Laminin or CD31, red) and/or the internal nerves (NF200, green). **I**–**P** Extracutaneous melanocytes in Iberian ribbed newt (*Pleurodeles waltl*) and in Eastern red-spotted newt (*Notophthalmus viridescens*) (**Q**–**W**), with white arrowheads pointing at extracutaneous pigment cells. **I**–**W** Extracutaneous melanocytes accompany blood vessels (Laminin, red) and nerves (Tuj1, green) within the trunk (**I**). Melanocytes were often found associated with single nerve fibers in the perimysium (**J**), surrounding nerve bundles (**K**) as well as blood vessels (**L**). In the salamander trunk, extracutaneous melanocytes were detected in multiple structures, including dorsal root ganglia (**M**), muscle (**J**), nerves (**K**), adipose tissue and inside vertebral bones (**L**). Pigment cells were also present above the brain (within the meninges covering the brain), and on the surface of the heart (specifically, in the pericardium—panels **O**, **P**). **Q**–**S** Melanocytes are found along the blood vessels and form capillary-associated pigmented spots within the skin also in the Eastern red-spotted newt. **T** Dissected sciatic nerve shows associated pigmentation. **U**–**W** Immunohistological staining for vessels (COL IV, red) and nerve fibers (TUJ1, green) shows melanocyte association with both types of structures in eastern red-spotted newt. Red arrows point at the pigmented blood vessels in (**Q**–**W**) and white arrows point at the pigmented spots in the skin in (**Q**). Scale bars are 1 mm (**A**, **I**, **P**), 500 µm (**I**), 100 µm (**E**–**H**, **M**–**N**), 50 µm (**B**, **Q**–**S**), 25 µm (**J**–**L**, **O**, **T**–**V**) and 10 µm (**C**, **D**, **W**)

**Fig. 7:**
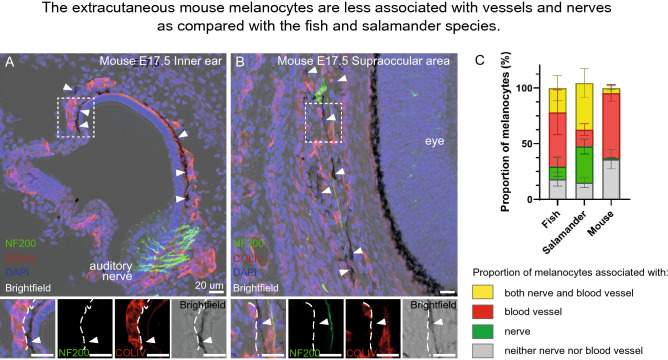
The extracutaneous mouse melanocytes are less associated with vessels and nerves as compared with the fish and salamander species. **A**, **B** Immunohistochemistry analysis for NF200 (green, marker of neuronal processes) and COLIV (red, marker of vessels) in a combination with brightfield images of pigmented melanocytes performed on sections through the inner ear (**A**, sagittal) and the suppraoccular region (**B**, coronal) of E17.5 mouse embryos. Melanocytes are pointed by the white arrowheads. Note the association of melanocytes with the blood vessels (marked by the white dashed line) shown in the insets. White dotted line marks the areas magnified in the insets. Scale bars are 20 µm. **C** Graph shows proportions of melanocytes (in %) associated with both nerves and blood vessels (yellow), blood vessels (red), nerves (green), neither nerve nor blood vessel (grey) in mouse, fish and salamander species. Note the differences in proportion of blood vessel- and/or nerve-associated melanocytes in mouse, fish and amphibians. Mean ± SEM in the following order: both nerves and blood vessels, blood vessels, nerves, neither nerve nor blood vessel are: fish (21.82 ± 5.70, 48.74 ± 9.98, 11.26 ± 4.27, 18.17 ± 3.10), salamander (41.93 ± 6.50, 15.17 ± 2.61, 32.43 ± 3.32, 14.99 ± 2.20), mouse (4.52 ± 0.93, 58.58 ± 3.73, 0.91 ± 0.32, 36.00 ± 4.37)

To test whether melanocytes associate with vessels and nerves in extracutaneous locations in anamniotes, we visualized vessels and nerves in fish and salamander species using immunohistochemistry. We detected their close association with pigmented and neuro-vascular cells and, thus, confirmed the wide-spread nature of nerve- and vessel-associated pigmentation in fish and amphibians (Figs. 6 and 7, Supplementary Figure 3). The conserved association of melanocytes with nerves and neuro-vascular bundles (Fig. 7) in anamniotes might suggest that nerve-associated glial cells could, in principle, convert into melanocytes similarly to how it happens in the case of a damaged mammalian nerve [32, 61]. Furthermore, the association of a significant fraction of melanocytes with blood vessels not accompanied by nerves (Fig. 7C, red bars) suggests that the vessels provide a niche and signaling support for melanocyte survival and expansion in fish and amphibians.

Taken together, these results point towards the ancient nature of SCP-to-melanocyte transition serving to populate extracutaneous locations with pigment cells. The internal pigmentation could contribute to the adaptation of animals to their habitats by boosting their camouflage, tuning physiological functions and protecting their internal organs from the UV radiation. Vessels, in this respect, could serve as an additional infrastructural niche facilitating the spread and survival of nerve-derived extracutaneous and cutaneous melanocytes.

## Materials and methods

### Mouse strains and genetic tracing

All animal experiments were approved by Ethik-Kommission zur Beratung und Begutachtung von Forschungsprojekten am Tier (Bundministerium fuer Wissenschaft, Forschung und Wirtschaft in Austria, BMWFW-66.009/0018-723, WF/V/3b/2017) and by Ethical Committee on Animal Experiments (North Stockholm Animal Ethics Committee, N226/15) and conducted according to the Austrian, Swedish and international guidelines. *Plp1*^*CreERT2*^ mice were cross-bred with a reporter mouse strain *Rosa26*^*YFP*^ [62]. Conditional *Ednrb* knockout mice have been described previously [59, 60]. Pregnant mice (bred as a combination of heterozygotes for both Cre-recombinase and YFP reporter) were injected with tamoxifen (Sigma, T5648; freshly dissolved in corn oil; administered dose 0.1 mg of tamoxifen/g of body weight) intraperitoneally at embryonic day E11.5, E12.5, E13.5, E14.5 or E15.5 to induce genetic tracing and dissected at E17.5. At least three embryos from independent litters were used for analysis in Fig. 1, and five embryos from three independent litters were analyzed in Fig. 2. Three wild-type and three *Ednrb* knockout embryos (each from three independent litters) were assessed during analysis (Fig. 3).

### Immunohistochemistry and whole-mount immunohistochemistry of mouse embryos

Pregnant mice were sacrificed by isoflurane (Baxter KDG9623) overdose. Dissected E10.5 or E17.5 embryos were shortly washed in PBS and fixed overnight in 4% paraformaldehyde (pH 7.4, in PBS), cryoprotected in 30% sucrose, embedded in OCT medium (Tissue-Tek, 4583) and sectioned at a thickness of 16 µm and placed on Thermo Scientific™ SuperFrost ™ Adhesion slides. Antigen retrieval (DAKO Target Retrieval Solution, S1699) was performed prior to the immunostaining in a steamer at a low pressure for 10 min. Sections were blocked for 20 min with Dako Real™ antibody diluent (S2022) and incubated with primary antibody overnight at + 4 °C. The next day, the primary antibody solution was collected and glasses with sections were washed in PBS, primary antibody mix was applied for additional 1 h staining at room temperature. Detection of primary antibody was performed with fluorophore-conjugated secondary antibodies (1:1000 from Invitrogen or 1:800 from Jackson Immuno Research) for 1 h at room temperature. Slides were mounted in 87% glycerol (Merck, 104094). Primary antibodies used for immunohistochemistry were: goat anti-GFP (Abcam, ab6662, 1:500), chicken anti-GFP (Abcam, ab13970, 1:500), chicken anti-GFP (Aves Labs Inc., #GFP-1020, 1:500), goat anti-SOX10 (R&D Systems, AF2864, 1:500), goat anti-MITF (R&D Systems, AF5769, 1:500), rabbit anti-COLIV (Biorad, 2150-1470, 1:1000), anti-CD31 (Abcam, ab28364, 1:100), anti-Laminin (Sigma Aldrich, L9393, 1:100) and chicken anti-NF200 (Abcam, ab4680, 1:1000).

Whole-mount immunohistochemistry of mouse embryos was performed as described previously [45]. For whole-mount immunohistochemistry, the following antibodies were used: goat anti-MITF (R&D Systems AF5769, 1:100), mouse anti-Neurofilament (Developmental Studies Hybridoma Bank 2H3, 1:100) and guinea pig anti-SOX10 (gift from Michael Wegner, Friedrich-Alexander-Universität Erlangen-Nürnberg, Germany; 1:500).

### RNAscope^®^ in situ hybridization

In situ hybridization using the RNAscope^®^ Fluorescent Multiplex Assay kit (version 1) was performed using pretreated cryosections of fixed frozen tissue according to the manufacturer’s instructions. Probes used are commercially available: Mm-*Mitf*-C2 (Cat No 422501-C2) and Mm-*Dct*-C3 (460461-C3).

### Immunofluorescent staining following RNAscope^®^ in situ hybridization

Following the complete hybridization protocol according to the manufacturer’s instructions, sections were incubated at 4 °C, overnight with primary antibodies diluted in 1×PBST (0.1% Tween‐20 in 1×PBS). Next, sections were washed in 1×PBST and incubated with secondary antibodies diluted in 1×PBST at room temperature for one hour. Following three washes at room temperature with 1×PBST, DAPI (Sigma, D9542) was applied on the sections at a concentration of 0.5 mg/mL and incubated for 5 min at room temperature. Lastly, the sections were washed three times in 1×PBST and mounted using Mowiöl mounting medium.

Primary antibodies used were chicken anti-GFP (Aves Labs Inc. #GFP-1020, 1:500) and goat anti-SOX10 (R&D systems #AF2864, 1:300).

For detection of the primary antibodies, secondary antibodies raised in donkey and conjugated with Alexa-488, -555 and -647 fluorophores were used (Molecular Probes, ThermoFisher Scientific, 1:1000).

### Newt and fish tissue

Eastern red-spotted newts (*Notophthalmus viridescens*) and Iberian ribbed newts (*Pleurodeles waltl*) were obtained from the colony established at Karolinska Institutet. Animal breeding and husbandry was performed according to standard protocols with minor modifications for the Eastern red-spotted newts [63]. Adult wild-type animals were used for the present study and sexes were considered separately for the analysis (*Notophthalmus viridescens*: *n* = 3 females and *n* = 3 males; *Pleurodeles waltl*: *n* = 5 females and *n* = 5 males). Newts were deeply anesthetized by immersion in 0.1% tricaine (MS-222, Sigma), weighted and measured prior to sacrifice. The tissue was fixed in 4% paraformaldehyde, dissected out, and imaged. Tissue processing and immunohistochemistry were performed as described previously [64]. Rabbit anti-COLIV (Biorad, 2150-1470, 1:1000), anti-CD31 (Abcam, ab28364, 1:100), anti-Laminin (Sigma Aldrich, L9393, 1:100-1:500) were applied to detect blood vessels; chicken anti-NF200 (Abcam, ab4680, 1:1000) and anti-TUJ1 (Promega, G712A, 1:500) were used to detect nerves in newts and fish. All procedures on newts were approved by the local ethics committee (Stockholms Djurförsöksetiska Nämnd) and were performed in accordance with national regulations issued by the Swedish Board of Agriculture. Pikeperch (*Sander lucioperca*), rainbow trout (*Oncorhynchus mykiss*) and European perch (*Perca fluviatilis*) were obtained from local fisheries and a local market.

### Image processing and data analysis

Images of YFP-traced samples and stained whole-mount embryos were taken using a Zeiss LSM880 confocal microscope and analyzed with Imaris software (Bitplane). Analysis of YFP positive/negative cells was performed in ZEN2.1 (ZEISS) software, cells were counted manually and at least 100 melanocytes per embryo were counted. Pictures of whole organs and salamander limbs were taken using Zeiss SteREO Lumar.V12 microscope with ZEISS Axiocam ERc 5 s Color Microscope Camera or a Leica M80 stereomicroscope equipped with a Leica IC80 HD camera. Images of newt melanocytes in hearts and brains were analyzed with ImageJ software. To study the association of melanocytes with nerves and/or blood vessels in *Pleurodeles waltl*, 5 trunk sections per animal containing between 387 and 891 melanocytes were quantified manually using ImageJ. Images of tissue sections stained with RNAscope^®^ in situ hybridization probes and immunohistochemistry of fish and salamander tissue sections were taken using Zeiss LSM700 and LSM980 confocal microscopes.

### Statistical analysis

Graphs and statistical analyses (two-way ANOVA; two-sided *t* test) were made using GraphPad Prism 8.3.0.

## Discussion

The extracutaneous melanocytes play several unconventional roles in the vertebrate body, including the key function of mediating the survival of the sensory hair cells in the inner ear [65], supporting the proper operation of the heart [66] or even facilitating the camouflage [67]. However, the specific origin of the extracutaneous melanocytes in the deep body locations has not been elucidated previously. Cells derived from different sources and having different biological history might reveal currently unknown variation in their properties and functions. Furthermore, short-living and long-living cell sources, together with their relative abundance in the body, are relevant for addressing the resulting tumors and other pathologies. Lastly, understanding the evolutionary mechanisms requires the knowledge of exact cellular sources of the investigated cell types and tissues. In case of cutaneous melanocytes, they can be derived immediately from the migratory neural crest cells or from the neural crest-derived SCPs covering early embryonic nerves [32, 45]. Therefore, the origin of extracutaneous melanocytes is starting to attract the attention of the research community.

Recently, several research teams have uncovered the mechanism of targeted recruitment of nerve-associated SCPs into various cell types both during embryonic development and in postnatal life [53–56, 68, 69]. The existence of committed, yet multipotent cells associated with the omnipresent body infrastructure such as nerves, represents a unique and accessible source of stem-like cells and an evolutionarily conserved mechanism that is evident in various species (for a comprehensive review, see [49]). Now, we report the targeted recruitment of SCPs into the fate of extracutaneous melanocytes.

In this study, we found that a significant portion of melanocytes located in different extracutaneous locations including the heart, brain meninges, supraorbital spaces and the inner ear are nerve derived. This result goes in line with the recent finding by Bonnamour et al., where the authors used a similar lineage-tracing strategy with glia-specific *Dhh-Cre* and *Plp1-CreERT2* lines to prove the predominant SCP-derived origin of cochlear, but not vestibular melanocytes of the inner ear [58]. Indeed, these intra-inner ear pigment cells are known for the essential role they play in the balance sensing and hearing: according to a number of studies, inner ear melanocytes condition the endolymph, which is necessary for the survival or development of the receptor hair cells. The endolymph is unusually rich in K^+^ ions, which is at least partly mediated by melanocytes of the *stria vascularis* (also known as intermediate cells) [9, 70]. At the same time, the function of other melanocytes in the inner ear stays rather unknown [71–73].

According to our data, the population of melanocytes in the brain meninges is mixed in terms of their origin. A significant proportion is derived from the peripheral nerves during the development of meninges. We additionally identified SCP-derived extracutaneous melanocytes occupying the region between the skull bones and the eyeballs in the vicinity of the optic nerve (supraorbital location). The exact physiological role of these melanocytes in this near-ocular location or in the brain meninges is currently unknown and requires further investigation. Finally, nearly all melanocytes within the heart turned out to be SCP-derived according to the high proportion of the lineage tracing. According to the literature, these cells play a role in conducting the electric signals within the cardiac muscle, and provide the stiffness to the cardiac valves [12–14]. Altogether, these results suggest that SCP-based nerve-dependent pathway of melanocyte generation has a varying power in different body locations. The joint contribution of SCP- and neural crest-derived melanocytes might provide the secure and highly tunable source of pigment cells in a plethora of extracutaneous locations.

In the context of evolution, the non-mammalian vertebrates also demonstrate a prominent association of pigment cells with various tissues deep inside the body (for a broad overview, see [74]). In fish and amphibians, melanophores occupy, apart from the skin, various connective tissues such as outer vascular walls, muscles, the epineurium, peritoneum and mesentery [75]. As it stands today, multiple studies performed in zebrafish established the ventral nerve-associated path of the neural crest dispersal, which might effectively lead to populating the extracutaneous locations with melanocytes. The corresponding neural crest-derived nerve-dwelling cells are likely homologous to mammalian SCPs and might be responsible for extracutaneous and even cutaneous pigmentation in lower vertebrates as discussed previously [33, 36, 50, 76]. Similarly, in avians, melanocytes also populate extracutaneous visceral connective tissues, periostea, muscles, ovaries and the peritoneum [77]. Here, we explored the extracutaneous locations analogous to those we analyzed in mice and found that in such extracutaneous locations the pigment cells are often associated with nerves and blood vessels. As vessels do often travel with nerves forming neuro-vascular bundles, it might require further investigation of the signaling mechanisms standing behind the vascular association with pigmented cells. Indeed, despite the fact that the nerves and neuro-vascular bundles showed conserved and systemic pigmentation within the bodies of anamniotes, we also noted the association of internal melanocytes with non-innervated blood vessels. This suggests that vessels might be the source of niche-forming signals allowing the nerve-derived melanocytes to multiply and spread through the entire body.

The signaling mechanisms responsible for the melanocyte recruitment from the innervation are not fully understood. However, there are natural clues, which enable better understanding of the situation. Silky chicken (Silkie fowl) is a remarkable example of the conserved molecular systems that drive the emergence of melanocytes and rely on Endothelin signaling [78–80]. In Silky chicken, duplication of the *Endothelin 3* (EDN3) gene causes internal body hyperpigmentation and results in melanocytes residing in muscles, cartilage, bone and connective tissues. Additionally, Waardenburg syndrome type 4 patients with a mutation in the endothelin-B receptor genes suffer from deafness and have a severe reduction of melanocytes in the inner ear [81]. Since endothelins and their receptors are expressed in SCPs of peripheral nerves, it can be presumed that such hyperpigmentation is a result of enhanced targeted recruitment of glial precursors into pigment-producing cells.

Furthermore, endothelins and their receptors play a negative role in the SCP to SC transition [82]. As we observed in our results, in the case of *Ednrb* knock out, in the region adjacent to the inner ear, the melanoblasts appear in a nerve-associated fashion in limited numbers and do not invade the inner ear. Since *Ednrb* is not convincingly expressed in murine melanoblasts [83] and is clearly found in SCPs, this result supports the importance of SCP-dependent origin of melanocytes in proper development of the inner ear and hearing function [84–86]. At the same time, Shin et al. reported that *Ednrb* is required for dispersal of melanocytes [86]. However, if SCPs inefficiently convert into melanoblasts without EDN3 signaling, this dispersal defect might be explained by the issues with local SCP recruitment into the pigment cell fate. In line with this, in the case of *Ednrb* knock out, we observe that a few non-migratory melanoblasts always stay in a nerve-associated mode, which supports their SCP-dependent origin in the otic vesicle region. This is consistent with our early findings of the role of EDN3 signaling in conversion of SCPs near the otic vesicle into melanocytes [45]. In accordance with these findings, the “Spot” mutation in the *Nr2f1-A830082K12Rik* gene pair locus stimulates the premature maturation of glial progenitors and leads to the hypopigmentation including in the inner ear, altogether causing a Waardenburg syndrome type 4-like phenotype [87].

Next, the SCP-dependent origin of pigment cells is important for understanding the origin and properties of extracutaneous melanocytic tumors that do not manifest at the level of the skin [88]. Not only subcutaneous melanomas but also neurofibromas, melanocytic schwannomas, melanocytomas and other Schwann cell-based tumors that often show moderate-to-heavy levels of pigmentation are often found in close association with spinal ganglia and nerves (reviewed by [89, 90]). Extracutaneous malignant melanomas are one of the deadliest tumors of an unclear etiology. These rare neoplasias can be found almost anywhere in the body and are almost impossible to detect early enough for a possible surgical treatment. Interestingly, although extracutaneous melanomas are in most cases considered a metastasis, an increasing number of authors report cases of malignant melanomas revealed inside the heart, on the spinal cord, and in leptomeninges without a primary tumor being identified [91–94]. The presence of melanomas at the locations that, under normal conditions, host the extracutaneous melanocytes, the absence of identified primary tumor and certain gene expression similarities between melanoma cells and the Schwann cells suggest these extracutaneous melanocytic tumors might be either derived from the recruited Schwann cells in that particular location or directly originate from the extracutaneous melanocytes present physiologically in these locations. Melanocytic schwannomas, benign neoplasms derived from glial cells, also show strong expression of S100 and some subsets also express GFAP (glial fibrillary acid protein), both being markers typical for SCs [95, 96]. A subset of schwannomas is also pigmented, ranging from mild to heavy pigmentation and cells express typical melanocytic markers [97–99]. Cells within these neoplasms seem to be thus a hybrid, expressing both markers of SCs and melanocytes and, interestingly, are usually located near paraspinal ganglia or along the spinal or autonomic nerves [100]. In a disorder called Neurofibromatosis type 1, a heterogeneous population consisting of Schwann cells, neurons, fibroblasts, mast cells and endothelial cells forms benign tumors originating either from the peripheral nerves in the dermis (dermal neurofibromas) or from any nerve inside the body (plexiform neurofibromas) [101]. In some cases, hyperpigmentation overlying the neurofibroma or pigment-producing cells within the tumor is present. Nevertheless, it is not clear whether these are melanocytes or melanin-producing SCs [102, 103]. This uncertainty suggests that some internal melanocytic tumors might be derived from the committed nerve-associated glial lineage and points to the delicate balance between the fate of SCs and melanocytes. Finally, the SCP-dependent origin of extracutaneous melanocytes in the mouse opens up the possibility to recruit new melanocytes from the omnipresent innervation for the management of syndromes such as Waardenburg syndrome type 4 and the loss of hearing associated with other hypopigmentation disorders.

## Supplementary Information

Below is the link to the electronic supplementary material.

Supplementary Figure 1: The additional extracutaneous sites with pigmented melanocytes in the adult mouse. **﻿A, B** Sagittally opened spinal column (after the removal of the spinal cord) reveals a pigmented dorsal root ganglion (DRG) magnified in (**﻿B**). **﻿C, D** The extracted spinal cord is pigmented. Pigmentation is also found along the arteries supplying the spinal cord with blood (D).White dotted line marks the dorsal root ganglia, red dotted line outlines the spine and the yellow arrowheads point at the pigmented areas or at the individual pigmented melanocytes. Scale bars are 200 µm (**﻿C**), 100 µm (**﻿A, D**), 50 µm (**﻿B**) (TIFF 364 KB).

Supplementary Figure 2: Non-pigmented *Dct*^+^ melanocytes are abundant in the embryonic heart and the inner ear at E17.5. The presence of the pigment granules in *Dct*^+^ melanocytes in the inner ear (﻿A–D**﻿A–D**) and in the heart (**﻿E–H**) was assessed by using microscopy with transmitted light, RNAscope^®^ probe (*Dct*) and immunohistochemistry (SOX10 and YFP). Yellow arrowheads point at the *Dct*^+^ non-pigmented melanocytes. Yellow-black arrowheads point at the neighboring melanocytes containing melanin granules. Scale bars are 20 µm (TIFF 1888 KB).

Supplementary Figure 3: Association of extracutaneous melanocytes with vessels and nerves. **﻿A, B** Distances between melanocytes and the nearest blood vessels or nerves was measured in E17.5 mouse cochlea and supraorbital region. Mean ± SEM: mouse cochlea, distance from a melanocyte to a blood vessel was 16.05 ± 1.76 and to a nerve was 72.01 ± 4.72; mouse supraorbital location, distance to a blood vessel was 8.57 ± 0.55 and to a nerve was 39.20 ± 2.17. **﻿C** Graph represents the proportion of melanocytes associated with both nerves and blood vessels (yellow), blood vessels only (red), nerves only (green), neither nerves nor blood vessels (grey) in salamander (*Pleurodeles waltl*), E17.5 mouse embryo and fish (pikeperch and trout) in different internal locations. Mean ± SEM (in order: both nerves and blood vessels (yellow), blood vessels only (red), nerves only (green), neither nerves nor blood vessels (grey)): *Pleurodeles waltl* trunk (41.93 ± 6.50, 15.17 ± 2.61, 32.43 ± 3.32, 14.99 ± 2.19), murine cochlea (0.56 ± 0.56, 53.78 ± 2.52, 1.31 ± 0.78, 44.36 ± 2.43), murine supraorbital space (8.49 ± 1.63, 63.38 ± 5.29, 0.50 ± 0.50, 27.63 ± 6.33), fish (trout) heart (18.30 ± 2.28, 55.95 ± 2.98, 2.95 ± 1.58, 22.81 ± 1.96), fish (pikeperch) brain (21.72 ± 9.17, 41.41 ± 16.65, 22.51 ± 11.64, 14.36 ± 5.48), fish (pikeperch) muscle (20.29 ± 7.08, 64.06 ± 7.85, 2.22 ± 1.08, 13.43 ± 1.95). For each species and location, at least three individuals were assessed (TIFF 465 KB).

Supplementary file 4: Overview of the embryonic locations containing extracutaneous melanocytes. Melanocytes were detected using RNAscope^®^ probes (*Dct, Mitf*) and immunohistochemistry (SOX10 and YFP) combined with the transmitted light images. *Dct*^+^ melanocytes were found in the embryonic heart (in the vicinity or being associated with the cardiac valves—non-pigmented *Dct*^+^ cells), meninges of the brain (mainly located along the brain midline—mostly pigmented *Dct*^+^ cells), inner ear and suborbital locations. Cutaneous melanocytes of the skin showed the same pattern of the *Dct*^+^ signal and SOX10 immunoreactivity as melanocytes found in the extracutaneous locations. Combined RNAscope^®^ for *Dct* and *Mitf* on sections of the embryonic inner ear showed overlap of the two mRNAs, further confirming the use of *Dct* as a melanocytic marker (PDF 5835 KB).

## Data Availability

The datasets generated during and/or analyzed during the current study are available from the corresponding author on reasonable request.

## Abbreviations

SC: Schwann cell
SCP: Schwann cell precursor
NCC: Neural crest cell
Ednrb: Endothelin receptor B
EDN3: Endothelin 3
DRG: Dorsal root ganglion
