# Supplementary figures and images for: Nerve-associated Schwann cell precursors contribute extracutaneous melanocytes to the heart, inner ear, supraorbital locations and brain meninges
Source: Cell Mol Life Sci. 2021 Jul 18;78(16):6033–49. doi: 10.1007/s00018-021-03885-9 (PMC8316242; doi:10.1007/s00018-021-03885-9)

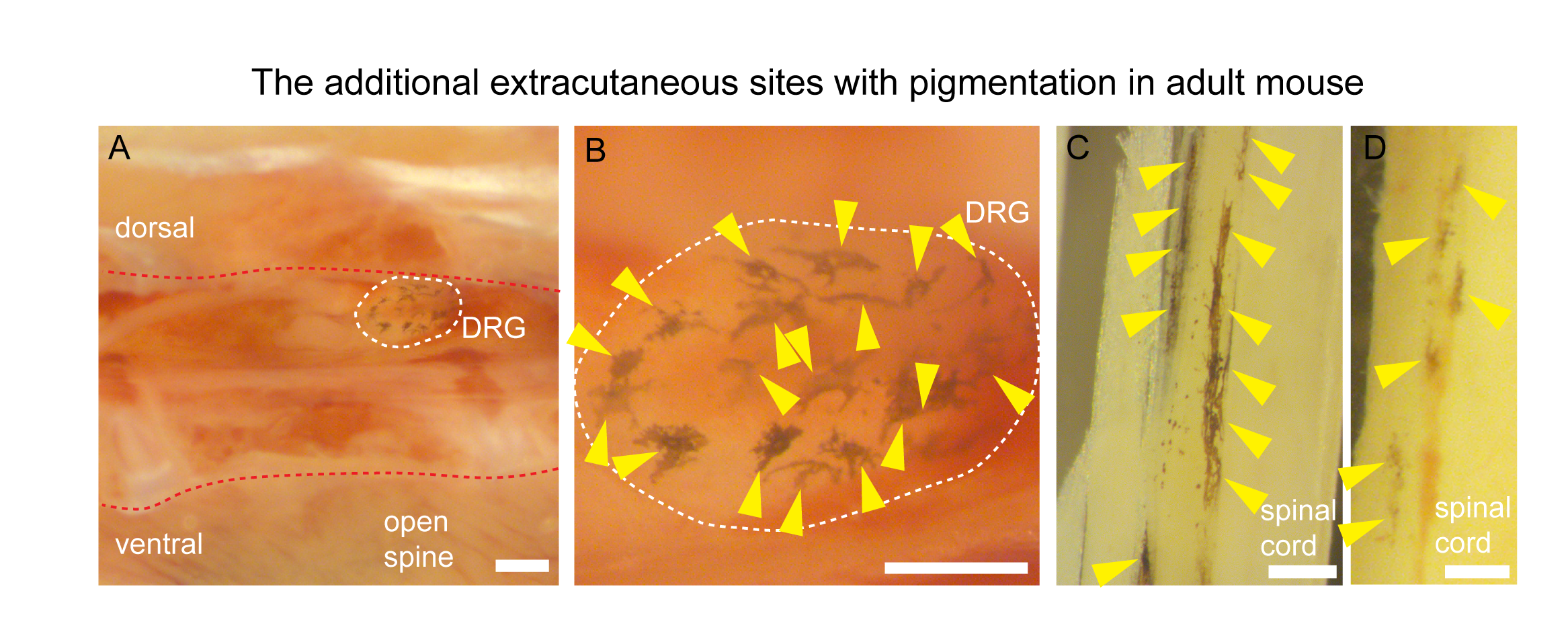

Supplement: Supplementary file 1 — Supplementary Figure 1: The additional extracutaneous sites with pigmented melanocytes in the adult mouse. ﻿A, B Sagittally opened spinal column (after the removal of the spinal cord) reveals a pigmented dorsal root ganglion (DRG) magnified in (﻿B). ﻿C, D The extracted spinal cord is pigmented. Pigmentation is also found along the arteries supplying the spinal cord with blood (D).White dotted line marks the dorsal root ganglia, red dotted line outlines the spine and the yellow arrowheads point at the pigmented areas or at the individual pigmented melanocytes. Scale bars are 200 µm (﻿C), 100 µm (﻿A, D), 50 µm (﻿B) (TIFF 364 KB). [file 18_2021_3885_MOESM1_ESM.tif]

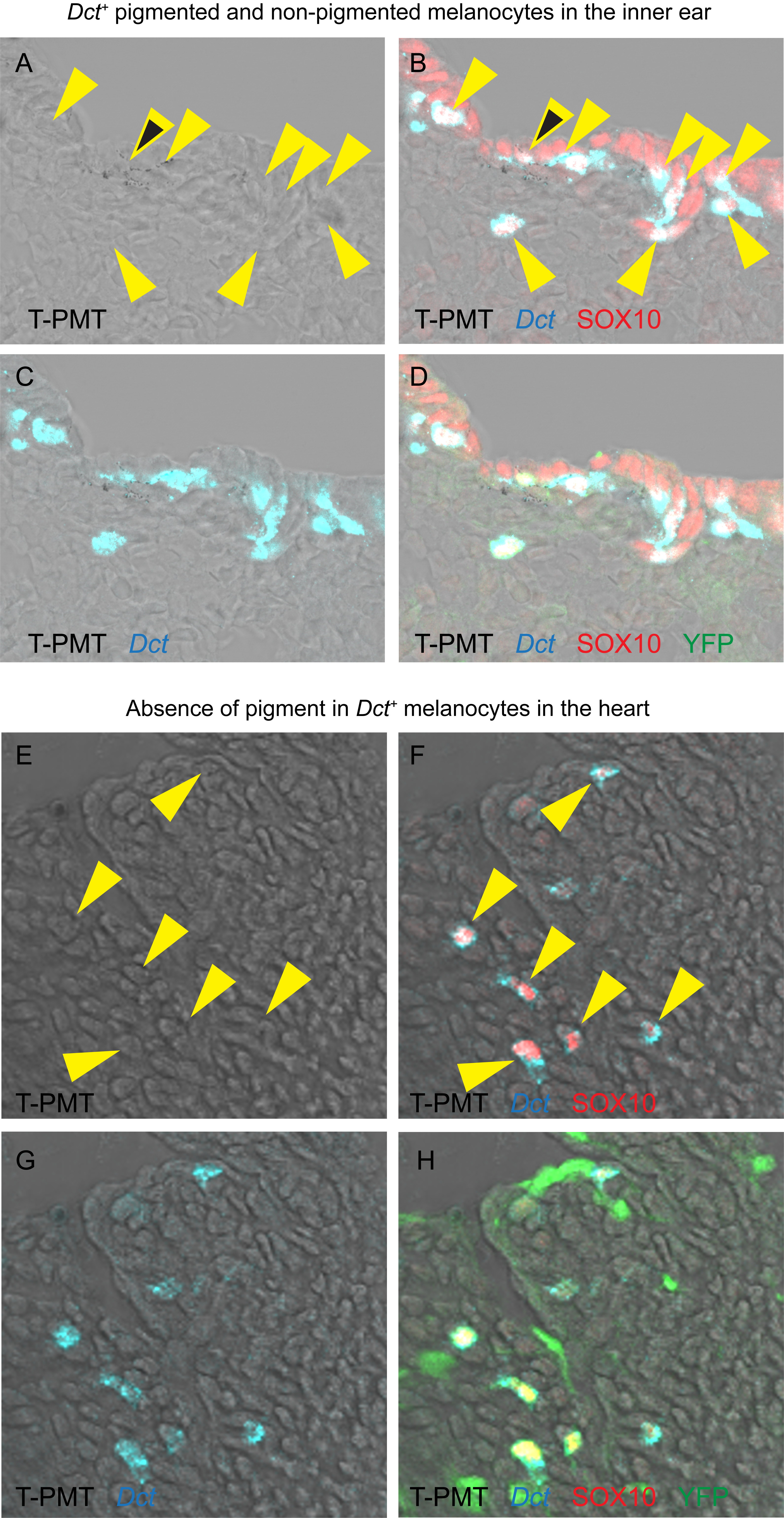

Supplement: Supplementary file 2 — Supplementary Figure 2: Non-pigmented Dct+ melanocytes are abundant in the embryonic heart and the inner ear at E17.5. The presence of the pigment granules in Dct+ melanocytes in the inner ear (﻿A–D﻿A–D) and in the heart (﻿E–H) was assessed by using microscopy with transmitted light, RNAscope® probe (Dct) and immunohistochemistry (SOX10 and YFP). Yellow arrowheads point at the Dct+ non-pigmented melanocytes. Yellow-black arrowheads point at the neighboring melanocytes containing melanin granules. Scale bars are 20 µm (TIFF 1888 KB). [file 18_2021_3885_MOESM2_ESM.tif]

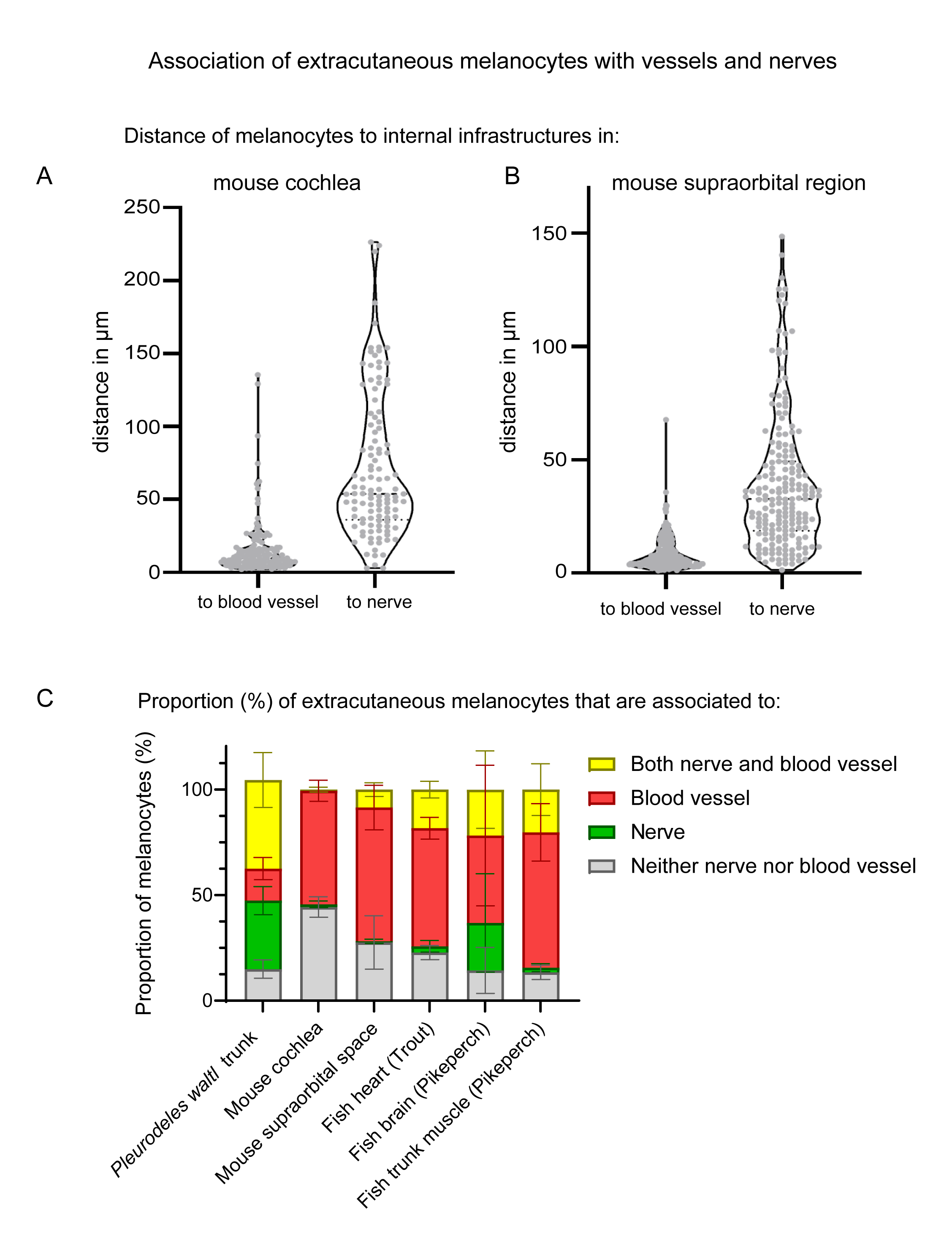

Supplement: Supplementary file 3 — Supplementary Figure 3: Association of extracutaneous melanocytes with vessels and nerves. ﻿A, B Distances between melanocytes and the nearest blood vessels or nerves was measured in E17.5 mouse cochlea and supraorbital region. Mean ± SEM: mouse cochlea, distance from a melanocyte to a blood vessel was 16.05 ± 1.76 and to a nerve was 72.01 ± 4.72; mouse supraorbital location, distance to a blood vessel was 8.57 ± 0.55 and to a nerve was 39.20 ± 2.17. ﻿C Graph represents the proportion of melanocytes associated with both nerves and blood vessels (yellow), blood vessels only (red), nerves only (green), neither nerves nor blood vessels (grey) in salamander (Pleurodeles waltl), E17.5 mouse embryo and fish (pikeperch and trout) in different internal locations. Mean ± SEM (in order: both nerves and blood vessels (yellow), blood vessels only (red), nerves only (green), neither nerves nor blood vessels (grey)): Pleurodeles waltl trunk (41.93 ± 6.50, 15.17 ± 2.61, 32.43 ± 3.32, 14.99 ± 2.19), murine cochlea (0.56 ± 0.56, 53.78 ± 2.52, 1.31 ± 0.78, 44.36 ± 2.43), murine supraorbital space (8.49 ± 1.63, 63.38 ± 5.29, 0.50 ± 0.50, 27.63 ± 6.33), fish (trout) heart (18.30 ± 2.28, 55.95 ± 2.98, 2.95 ± 1.58, 22.81 ± 1.96), fish (pikeperch) brain (21.72 ± 9.17, 41.41 ± 16.65, 22.51 ± 11.64, 14.36 ± 5.48), fish (pikeperch) muscle (20.29 ± 7.08, 64.06 ± 7.85, 2.22 ± 1.08, 13.43 ± 1.95). For each species and location, at least three individuals were assessed (TIFF 465 KB). [file 18_2021_3885_MOESM3_ESM.tif]
