# Supplementary material for: Nerve-associated Schwann cell precursors contribute extracutaneous melanocytes to the heart, inner ear, supraorbital locations and brain meninges
Source: Cell Mol Life Sci. 2021 Jul 18;78(16):6033–49. doi: 10.1007/s00018-021-03885-9 (PMC8316242; doi:10.1007/s00018-021-03885-9)
Supplement: Supplementary file 4 — Overview of the embryonic locations containing extracutaneous melanocytes. Melanocytes were detected using RNAscope® probes (Dct, Mitf) and immunohistochemistry (SOX10 and YFP) combined with the transmitted light images. Dct+ melanocytes were found in the embryonic heart (in the vicinity or being associated with the cardiac valves—non-pigmented Dct+ cells), meninges of the brain (mainly located along the brain midline—mostly pigmented Dct+ cells), inner ear and suborbital locations. Cutaneous melanocytes of the skin showed the same pattern of the Dct+ signal and SOX10 immunoreactivity as melanocytes found in the extracutaneous locations. Combined RNAscope® for Dct and Mitf on sections of the embryonic inner ear showed overlap of the two mRNAs, further confirming the use of Dct as a melanocytic marker (PDF 5835 KB). [file 18_2021_3885_MOESM4_ESM.pdf]

Low-magnification (20x objective) scan of the heart

- Sagittal section
- T-PMT (transmitted light, brightfield)
- GFP (green, antibody staining in the cytoplasm)
- SOX10 (red, nuclear staining using antibody)
- *Dct* (white, dotted pattern in the cytoplasm, RNAscope® probe)

Scale bars represent 500 µm

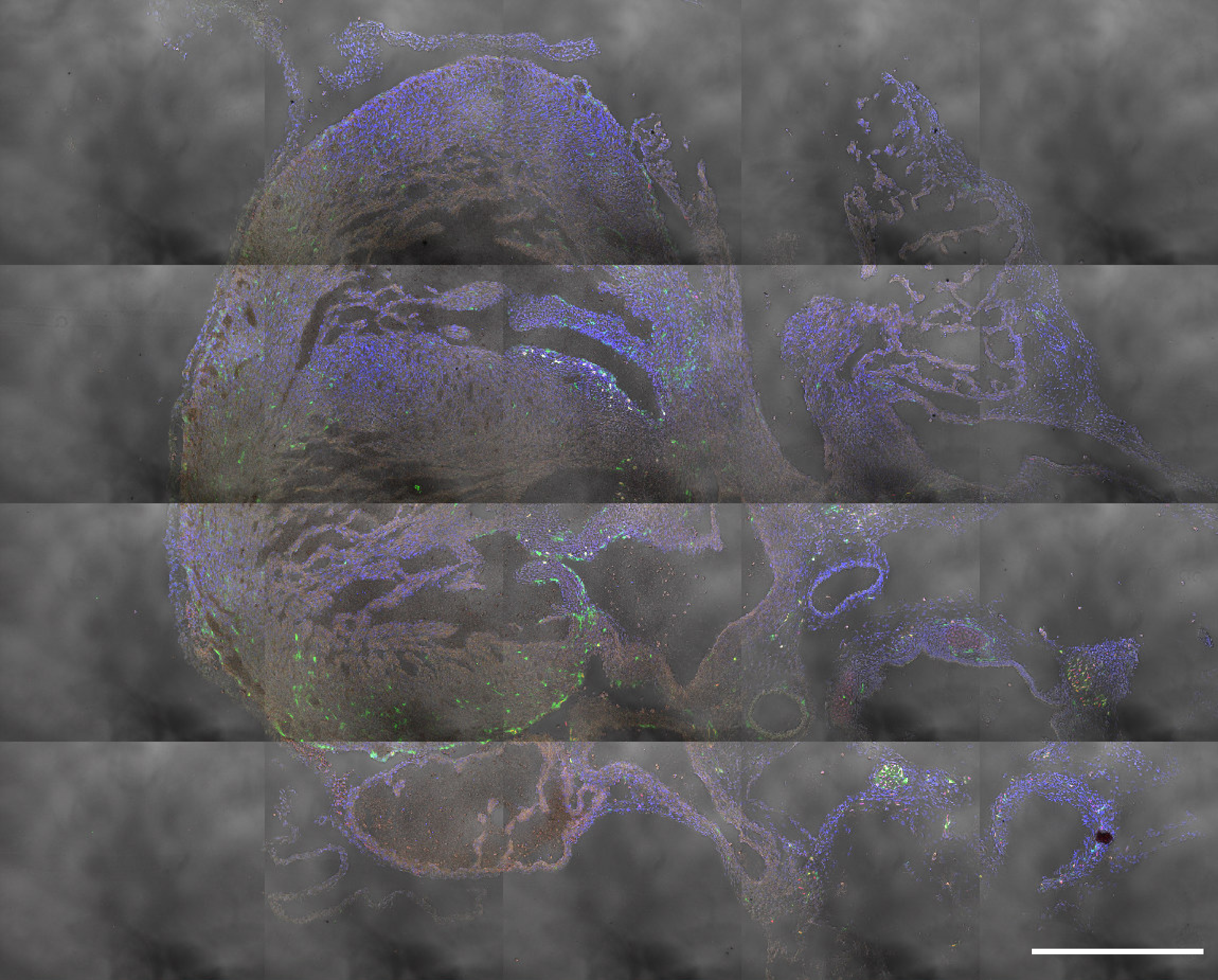

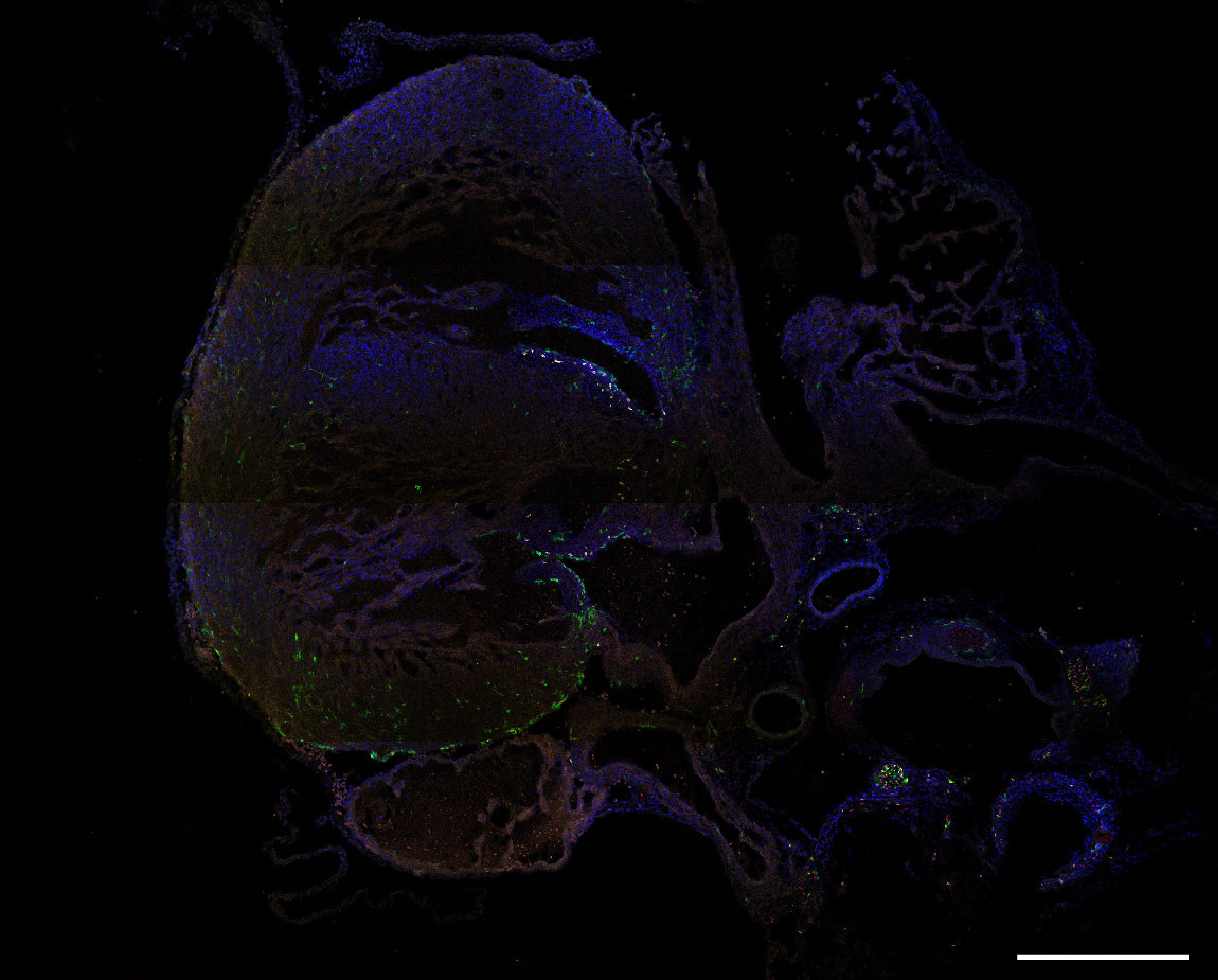

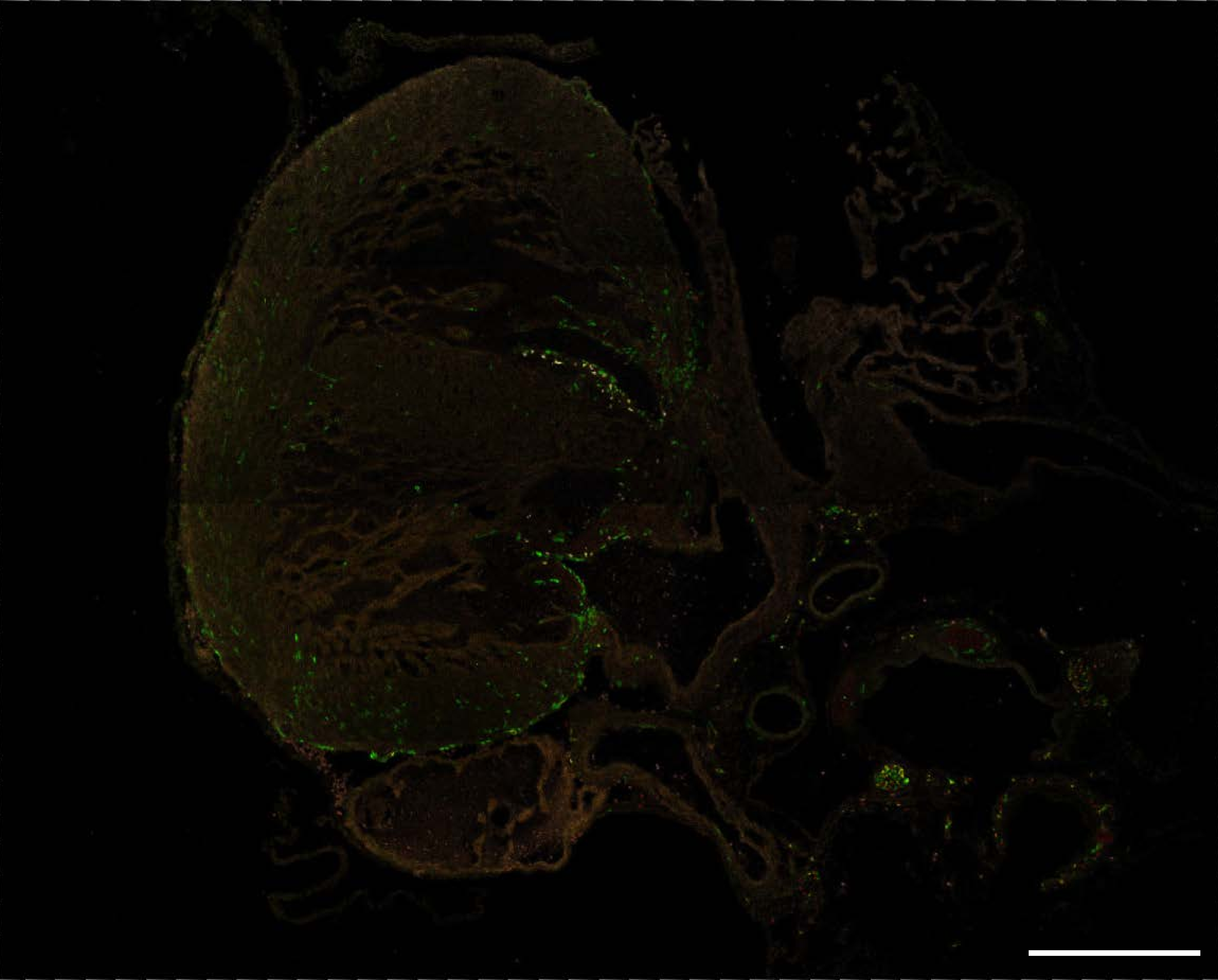

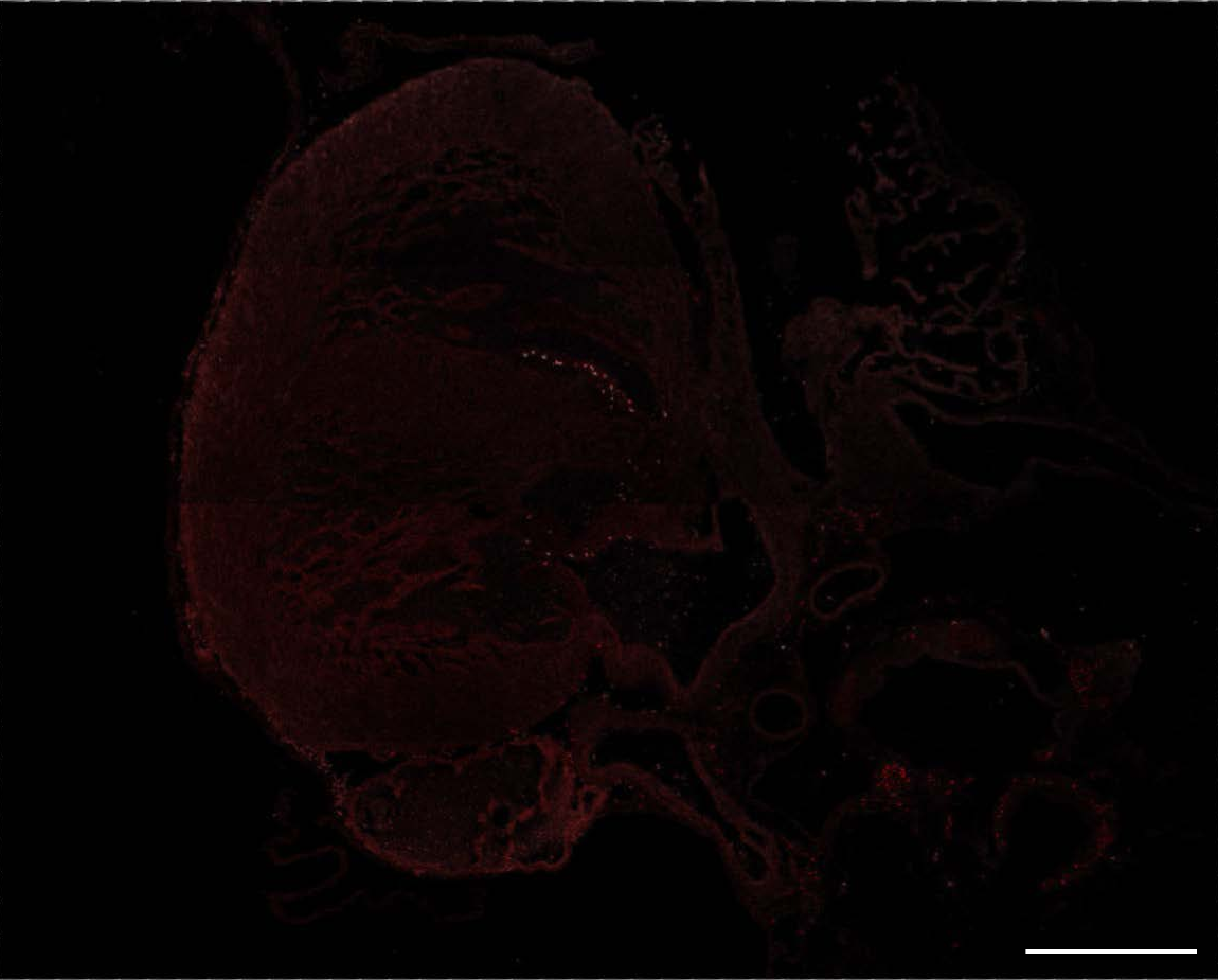

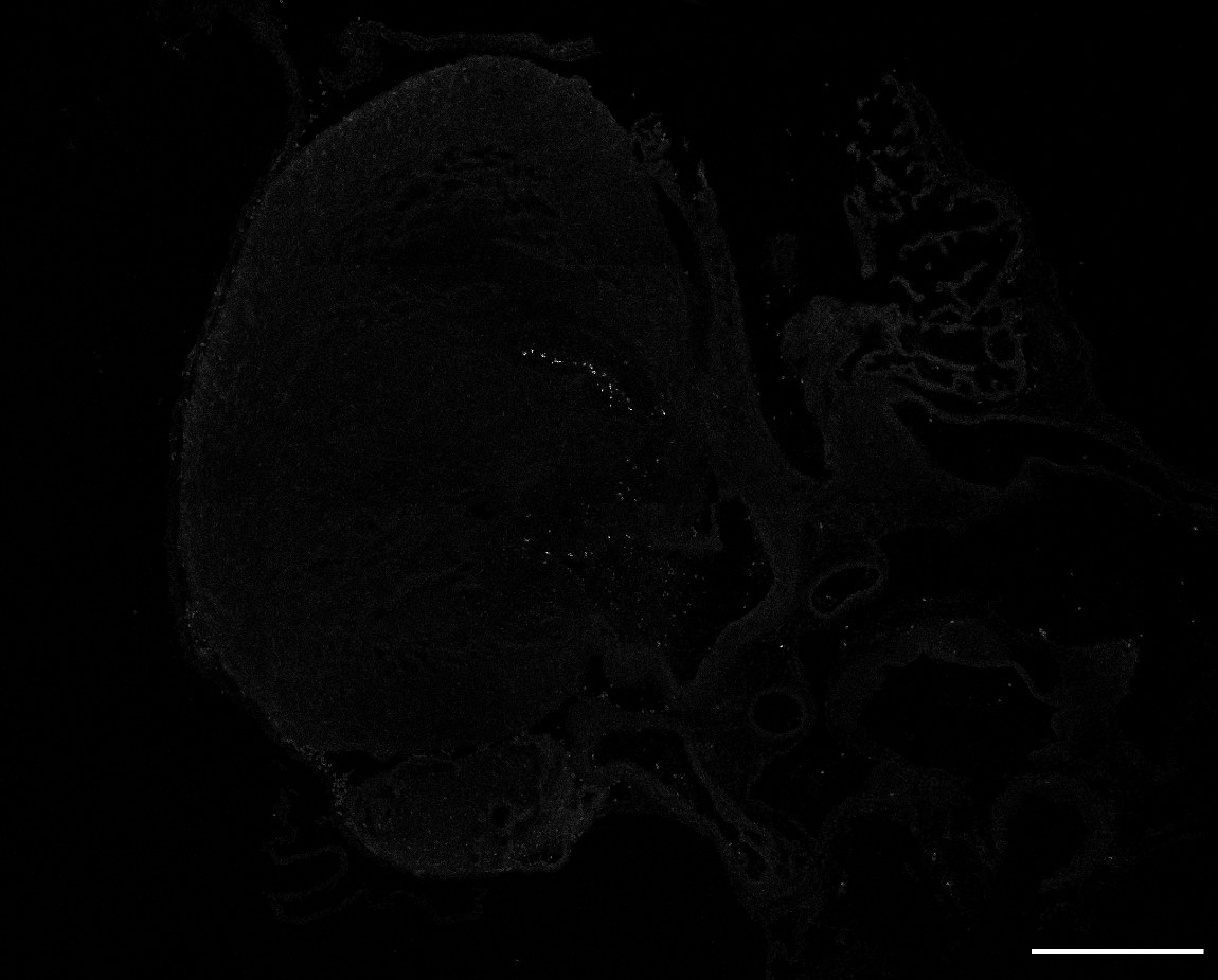

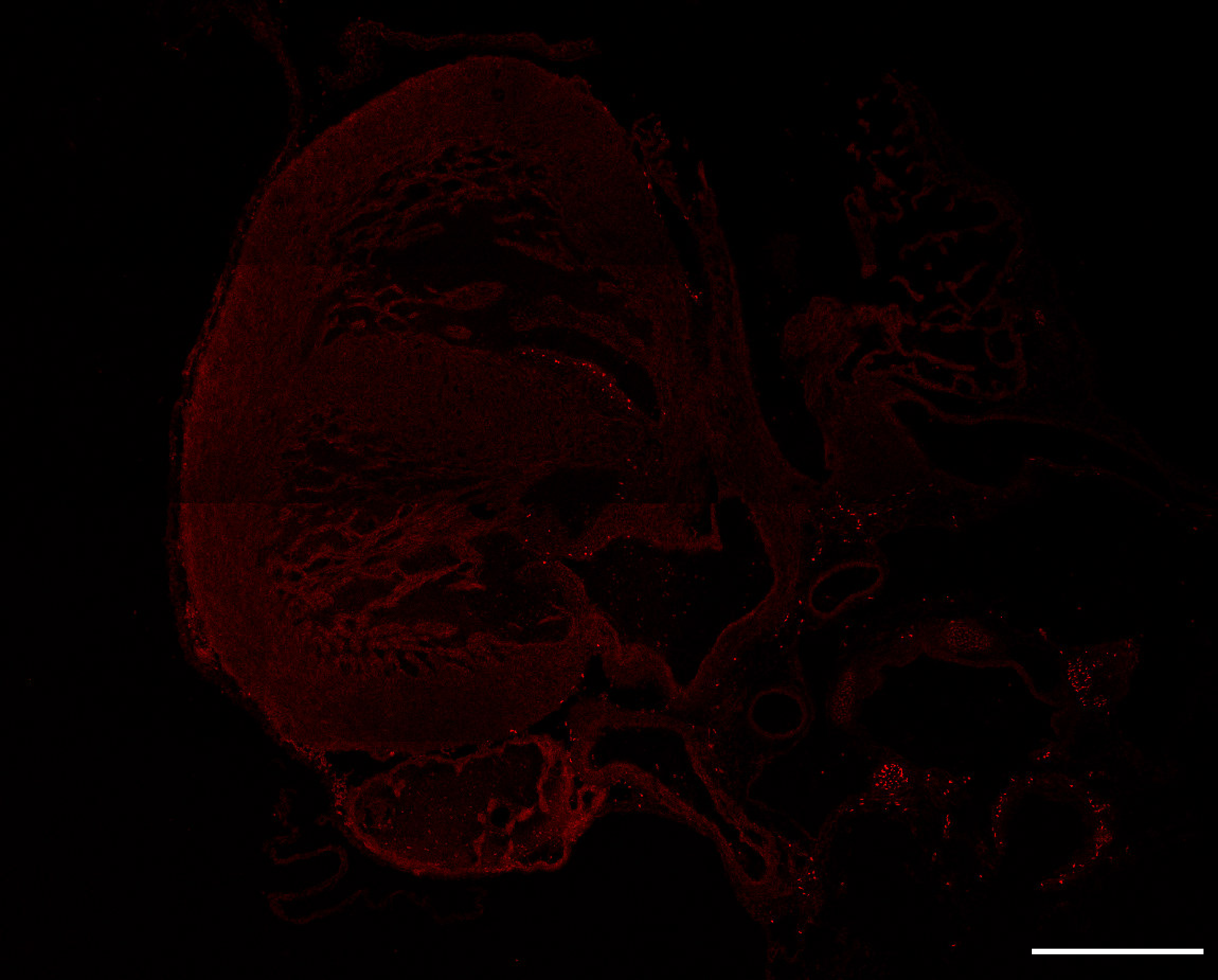

Low-magnification (20x objective) scan of the brain

- Horizontal section
- T-PMT (transmitted light, brightfield)
- GFP (green, cytoplasmatic, antibody staining)
- SOX10 (red, nuclear staining using antibody)
- *Dct* (white, dotted pattern in the cytoplasm, RNAscope® probe)

Scale bars represent 100 μm

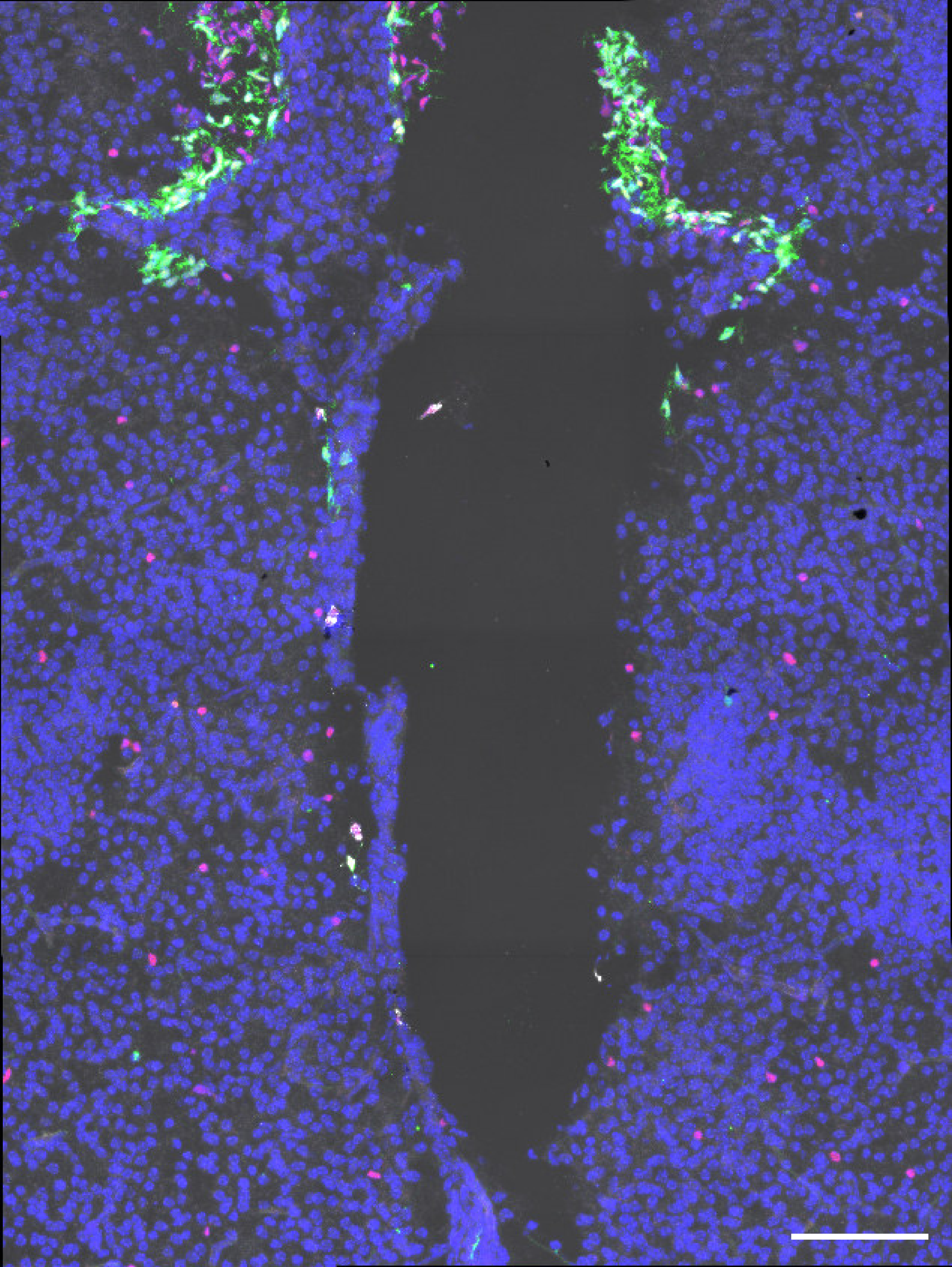

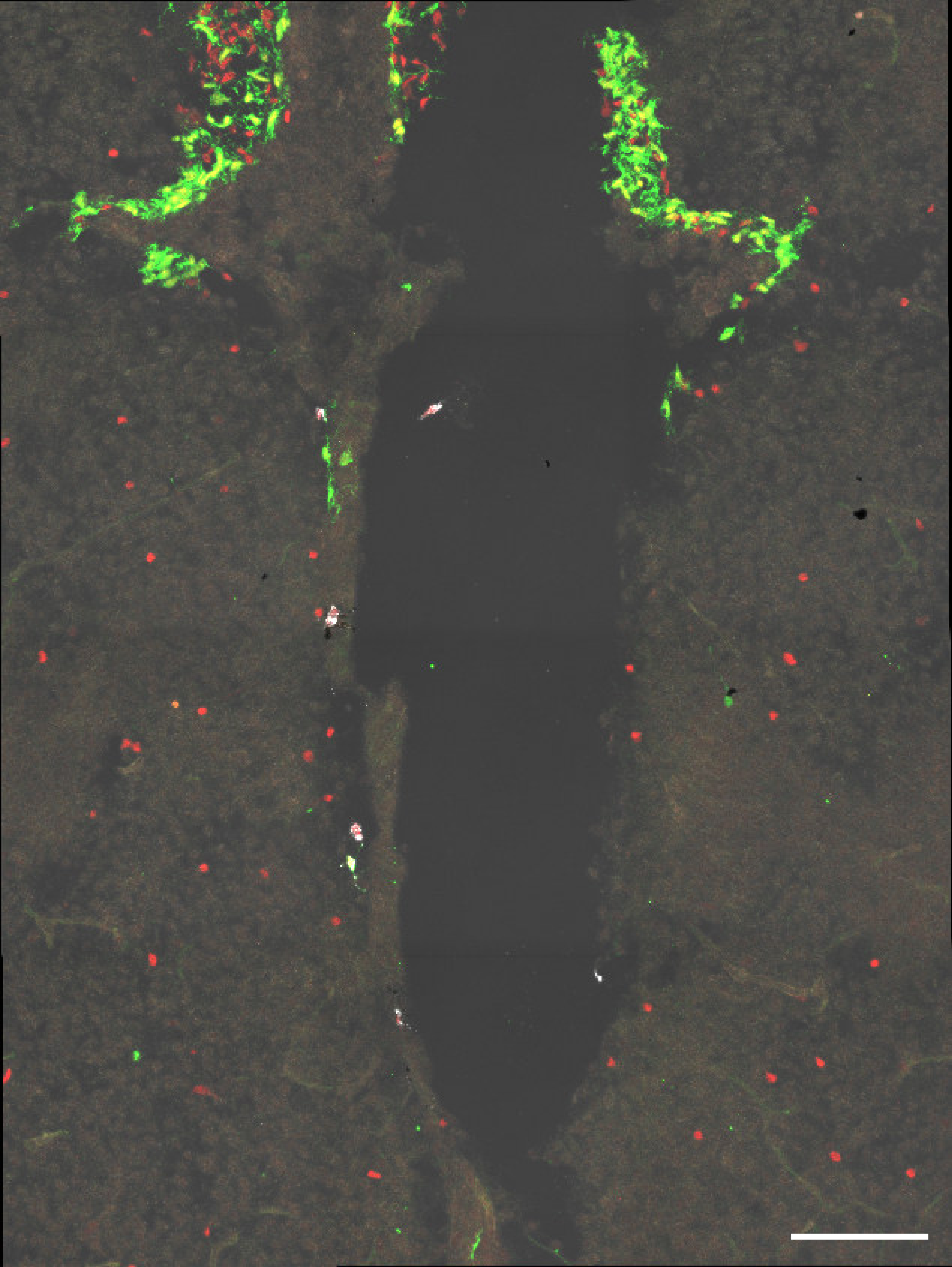

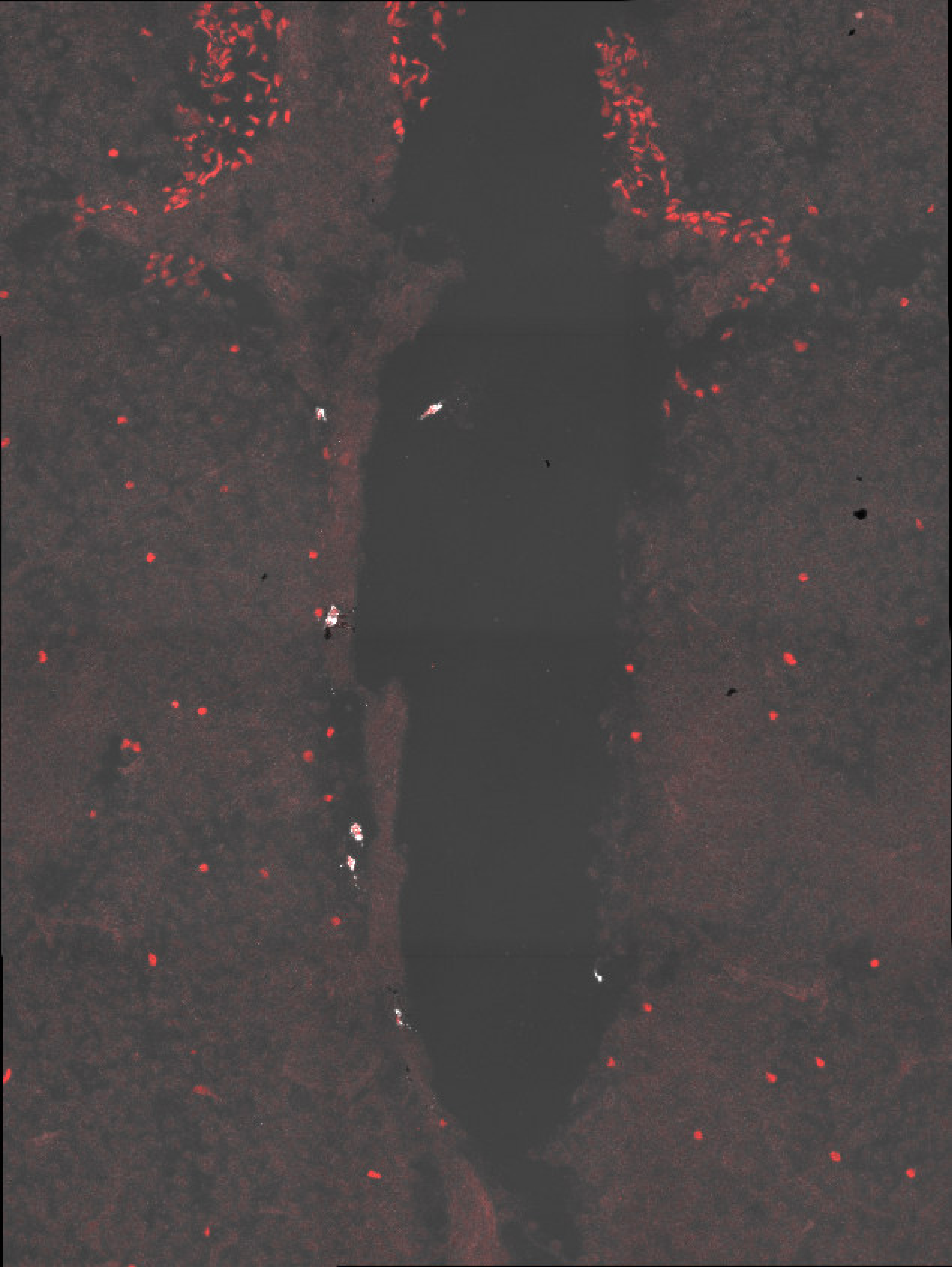

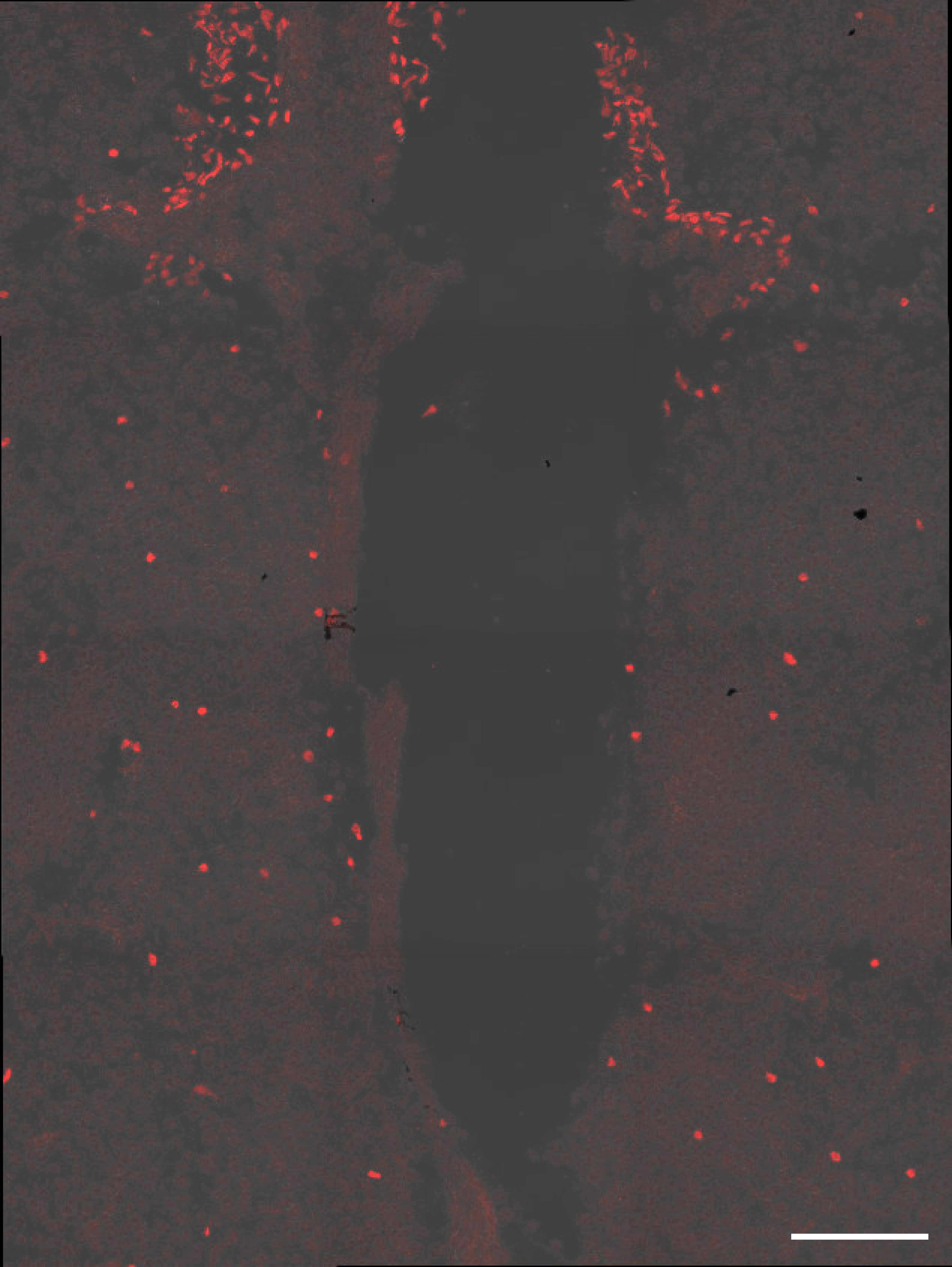

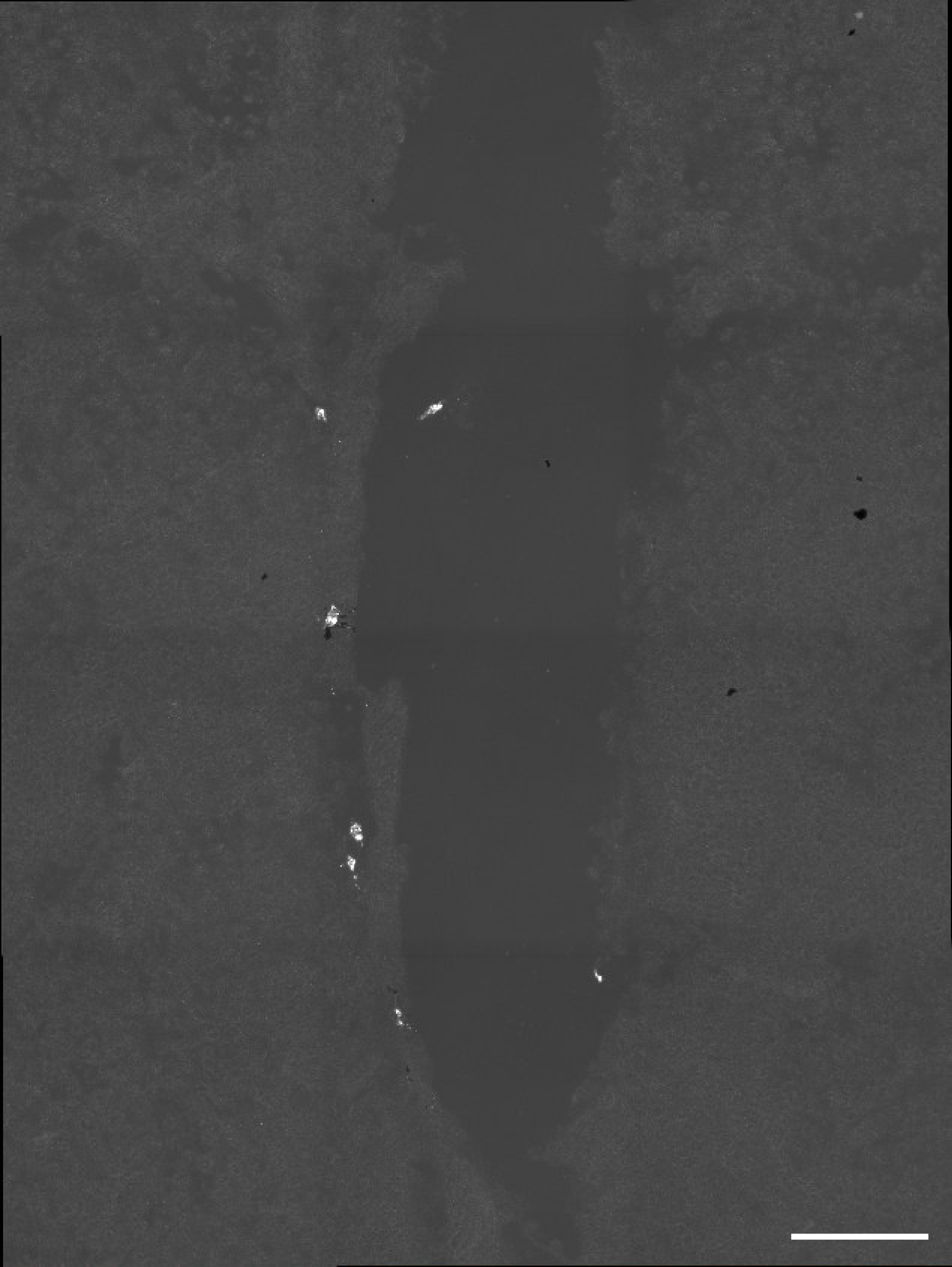

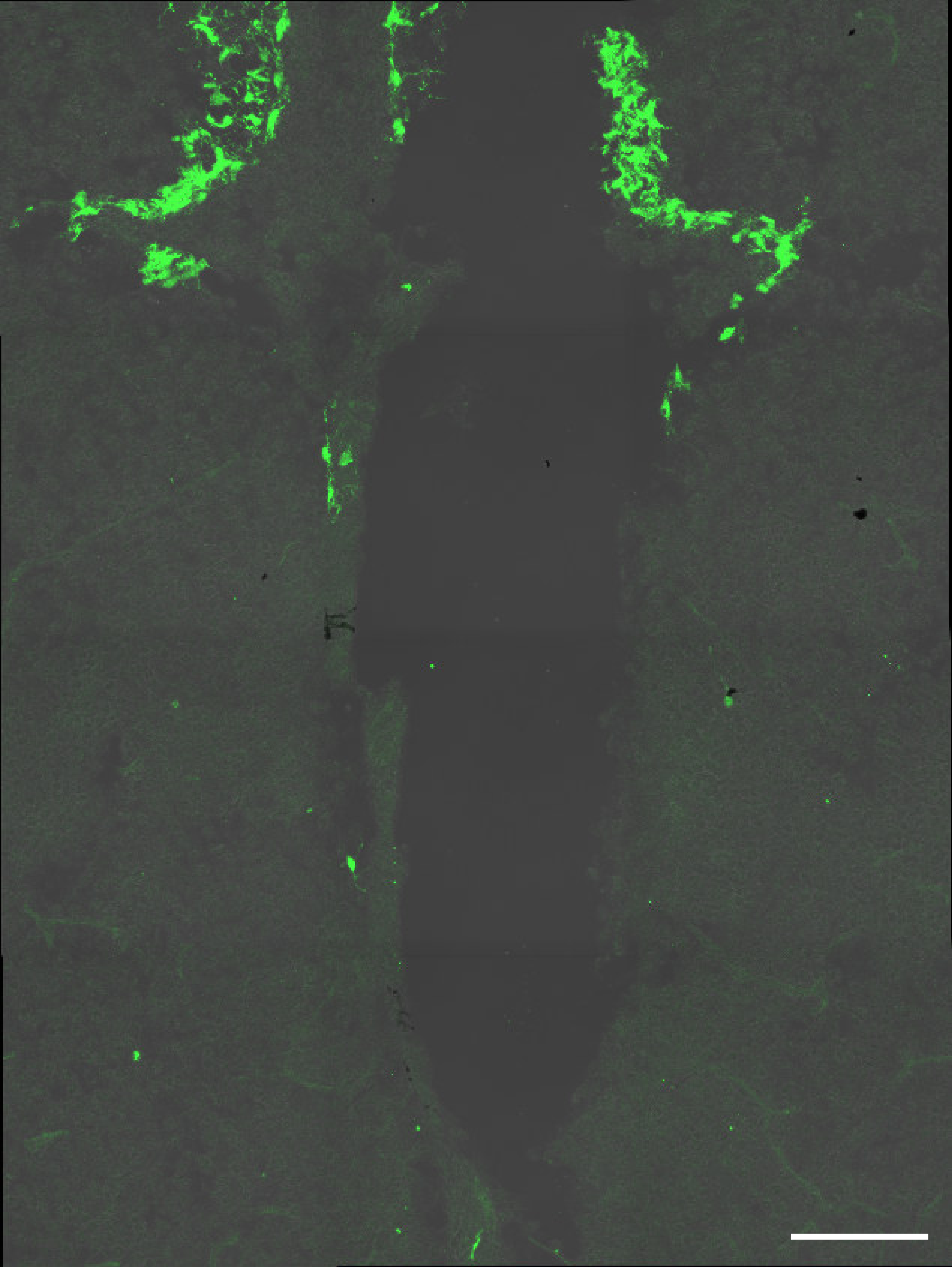

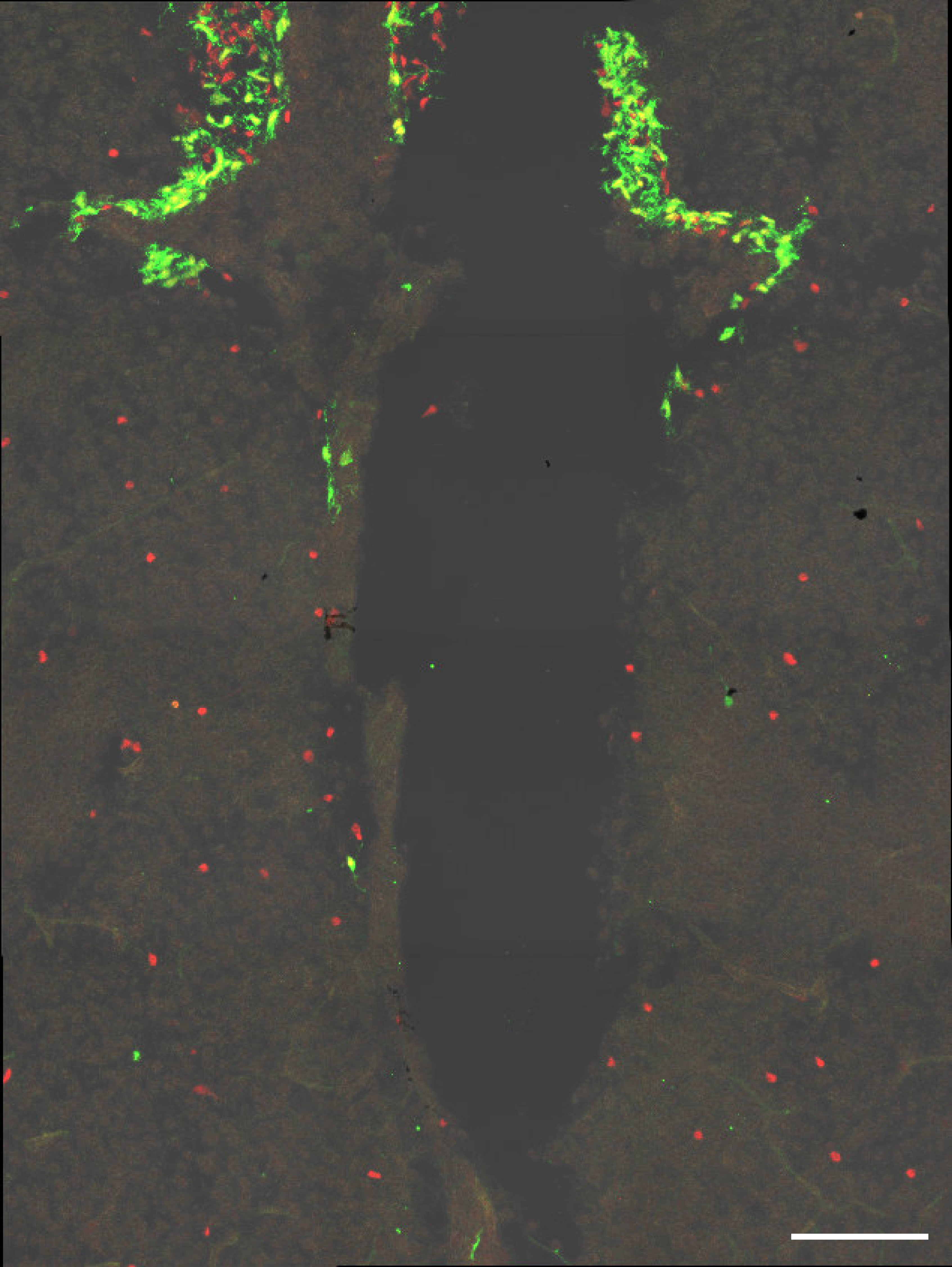

Low-magnification (20x objective) scan of the inner ear

- Sagittal section
- T-PMT (transmitted light, brightfield)
- GFP (green, cytoplasmatic, antibody staining)
- SOX10 (red, nuclear staining using antibody)
- *Dct* (white, dotted pattern in the cytoplasm, RNAscope® probe)

Scale bars represent 100 μm

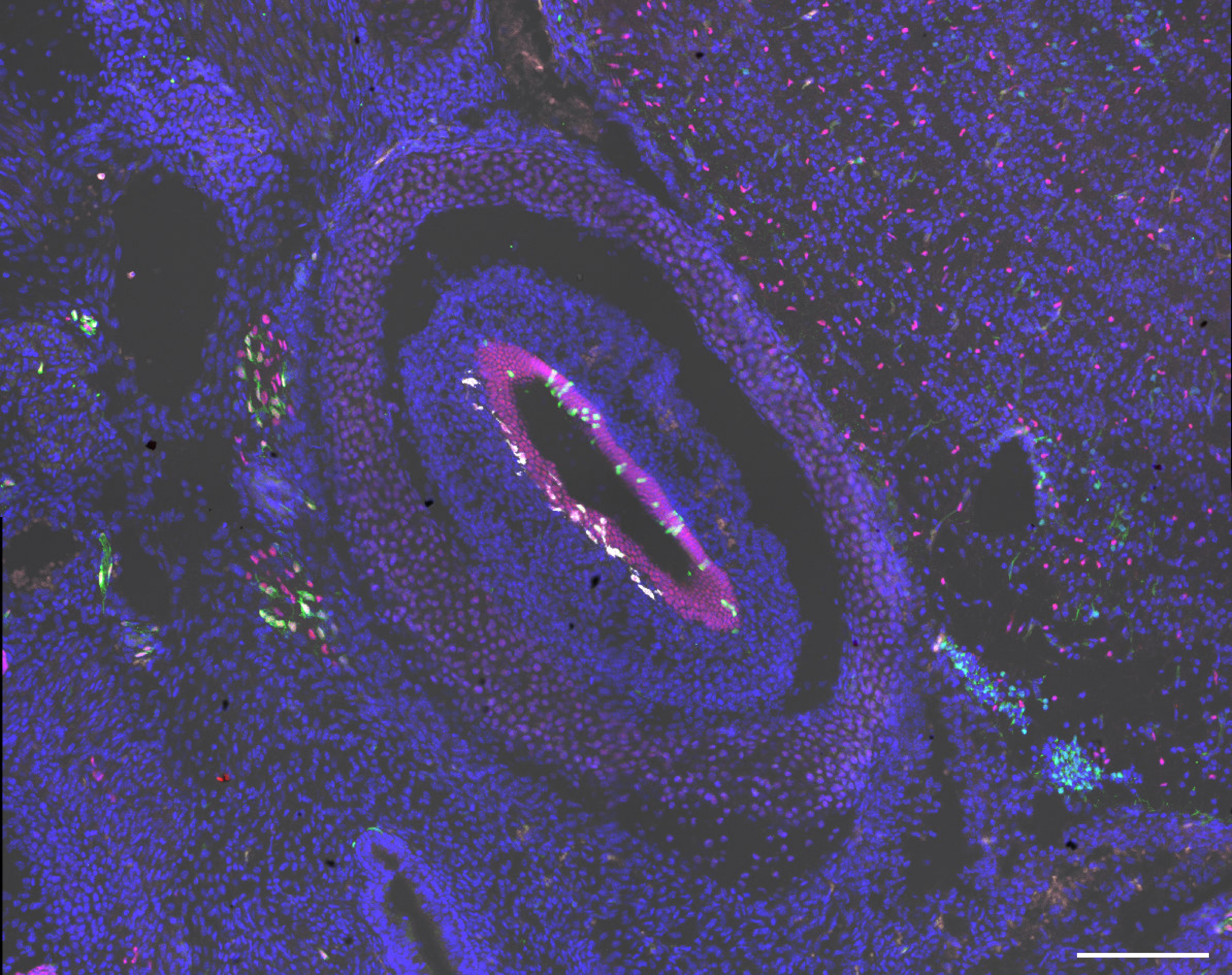

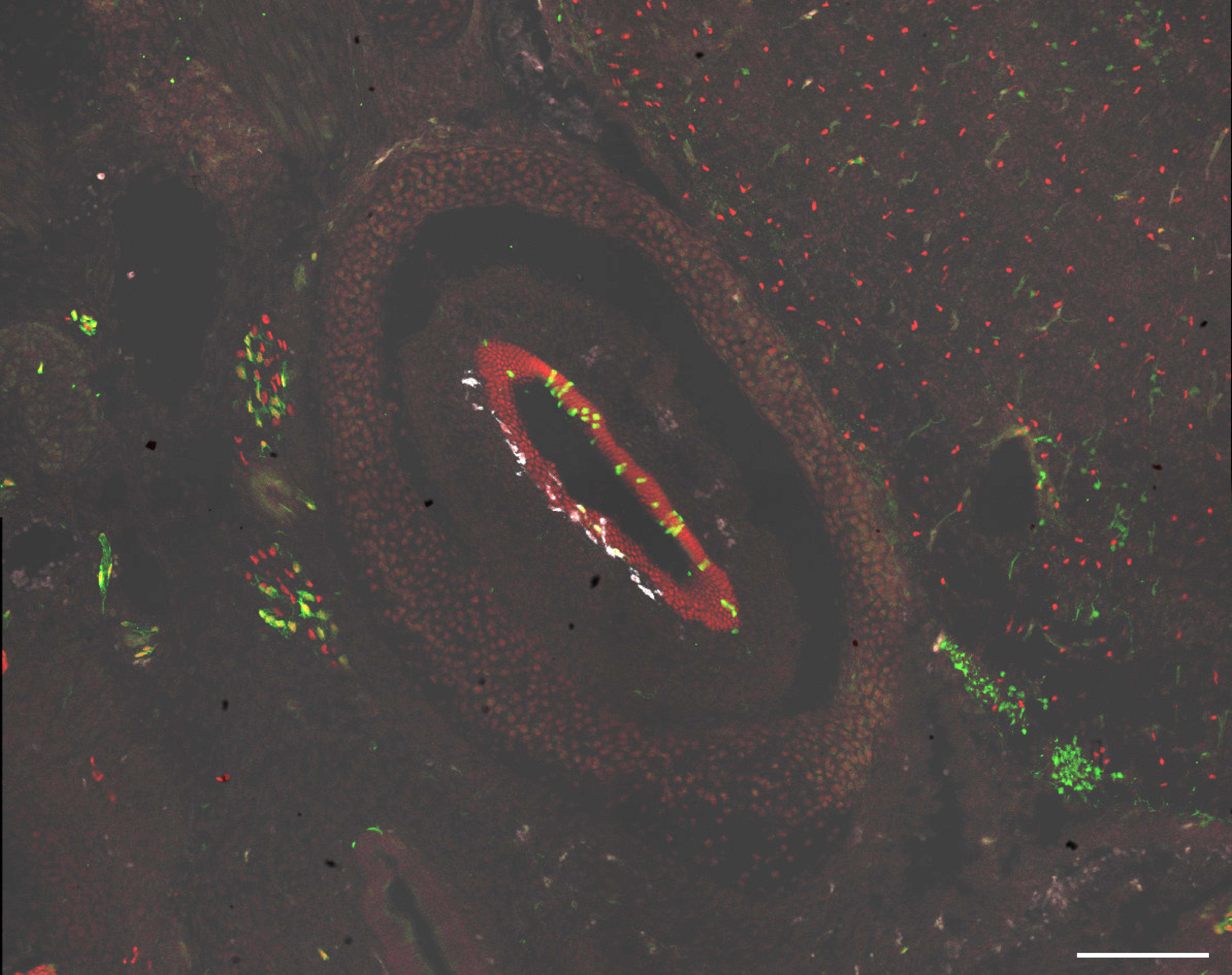

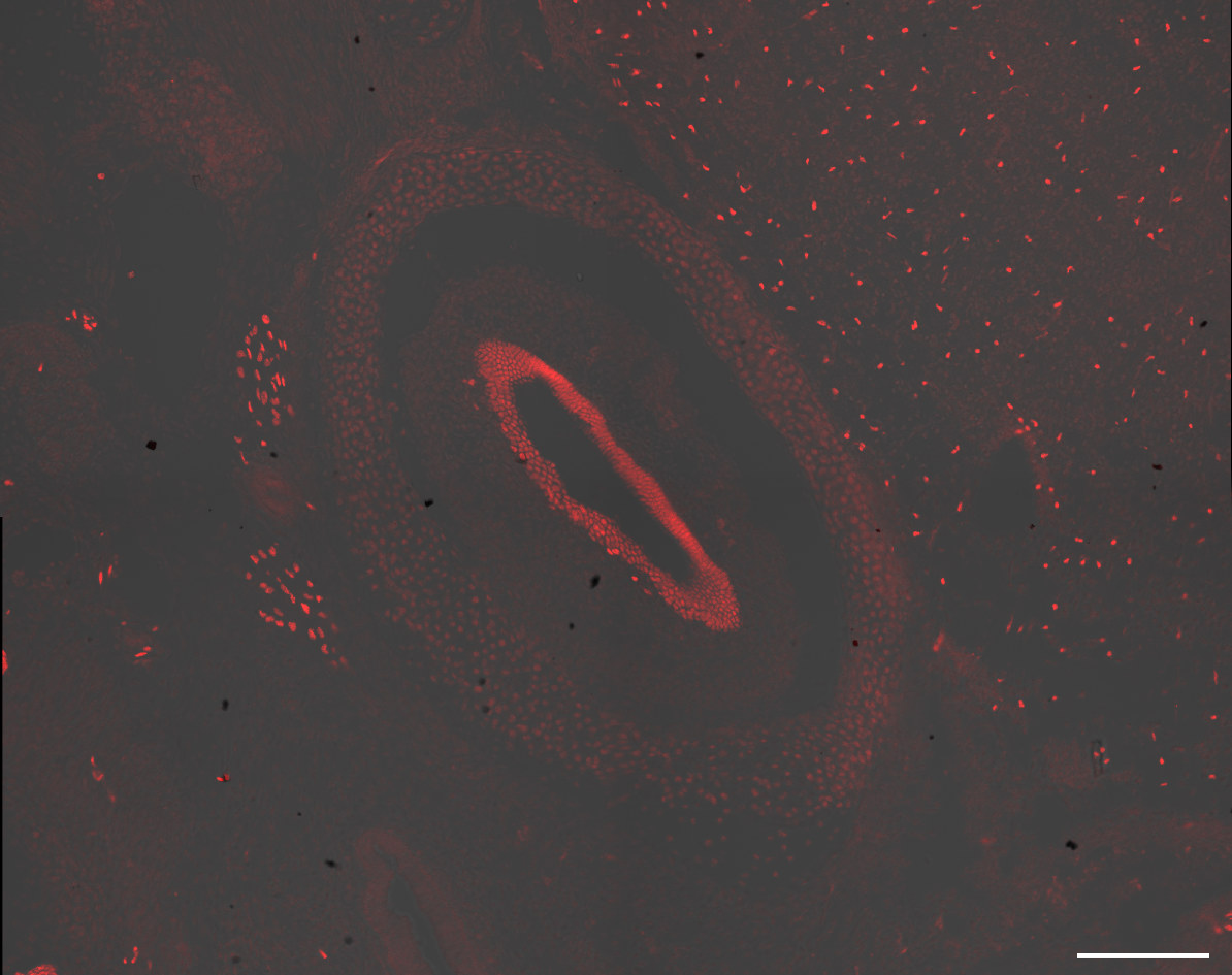

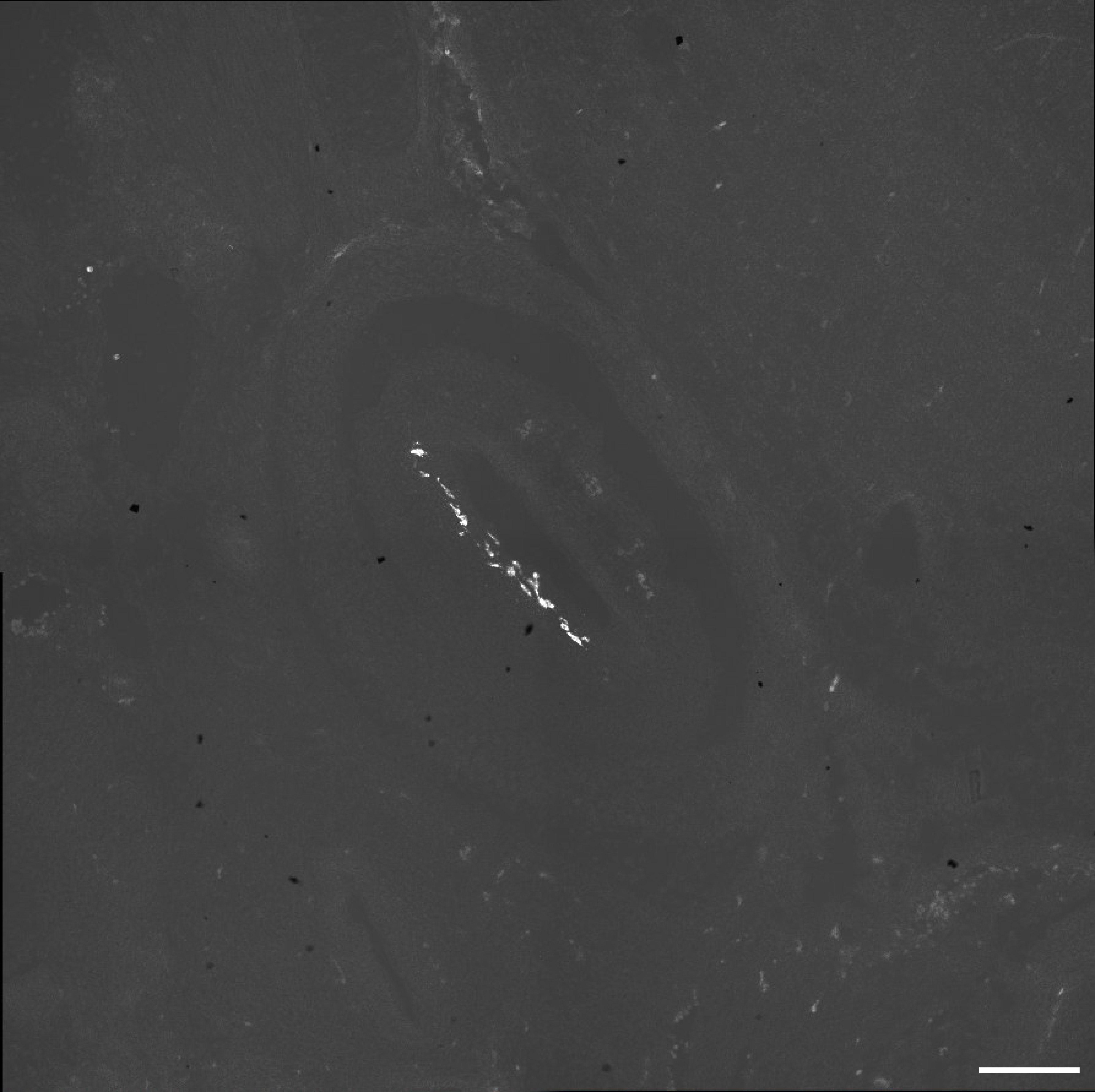

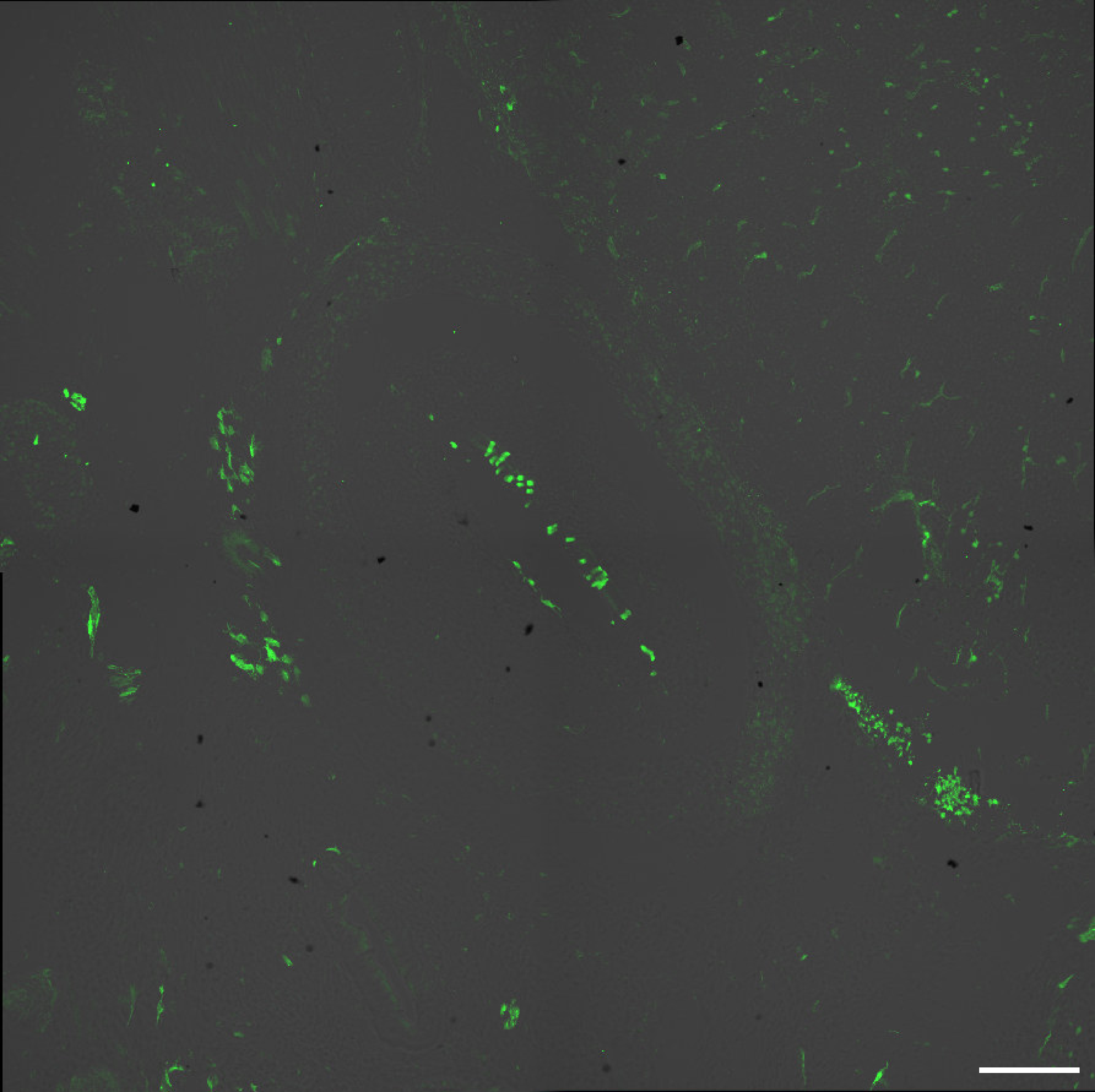

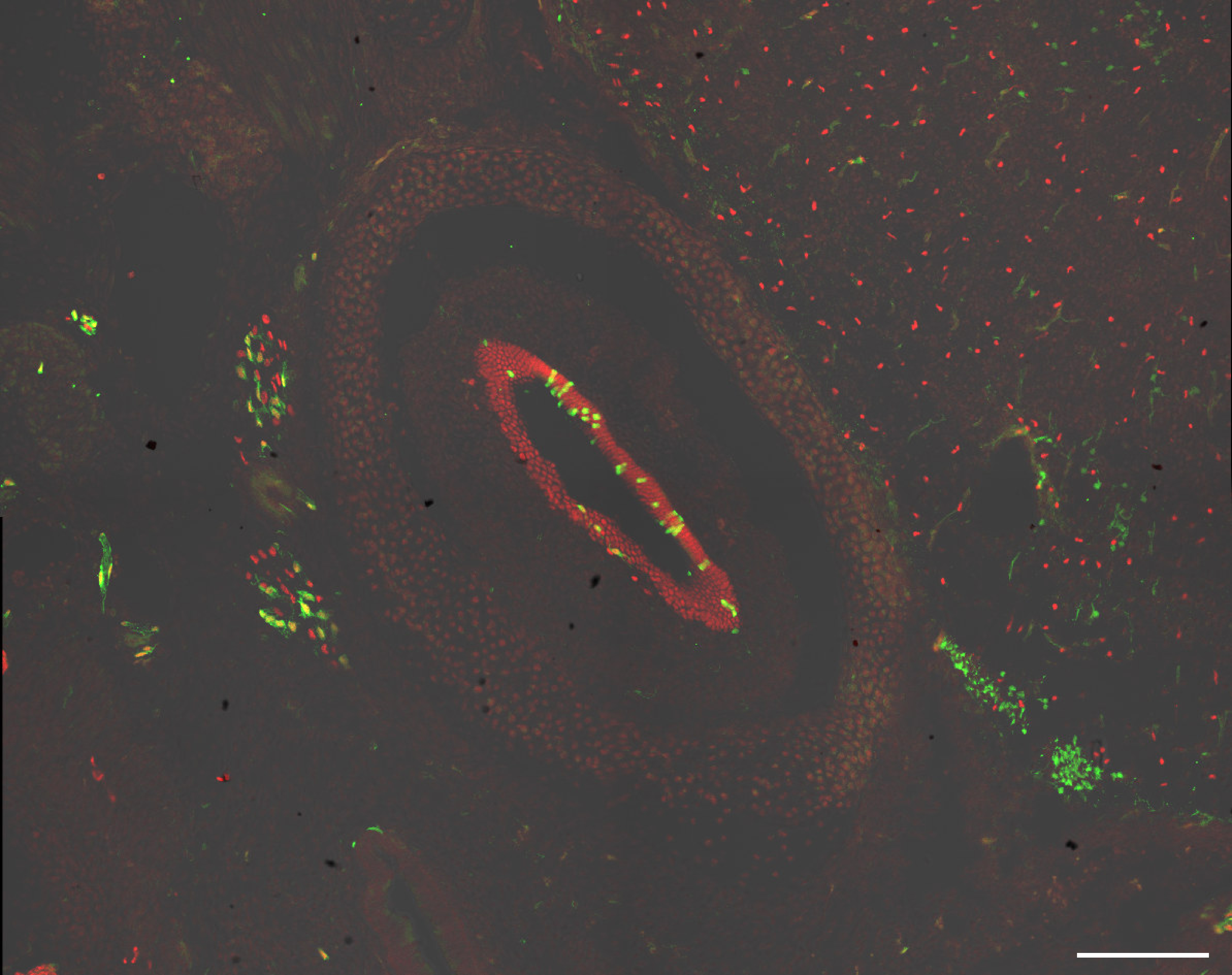

High-magnification (40x objective) scan of the inner ear – part 2 – colocalization of MITF/DCT

- Sagittal section
- T-PMT (transmitted light, brightfield)
- GFP (green, cytoplasmatic, antibody staining)
- *Mitf* (red, dotted pattern in the cytoplasm, RNAscope® probe)
- *Dct* (white, dotted pattern in the cytoplasm, RNAscope® probe)

Scale bars represent 100 µm

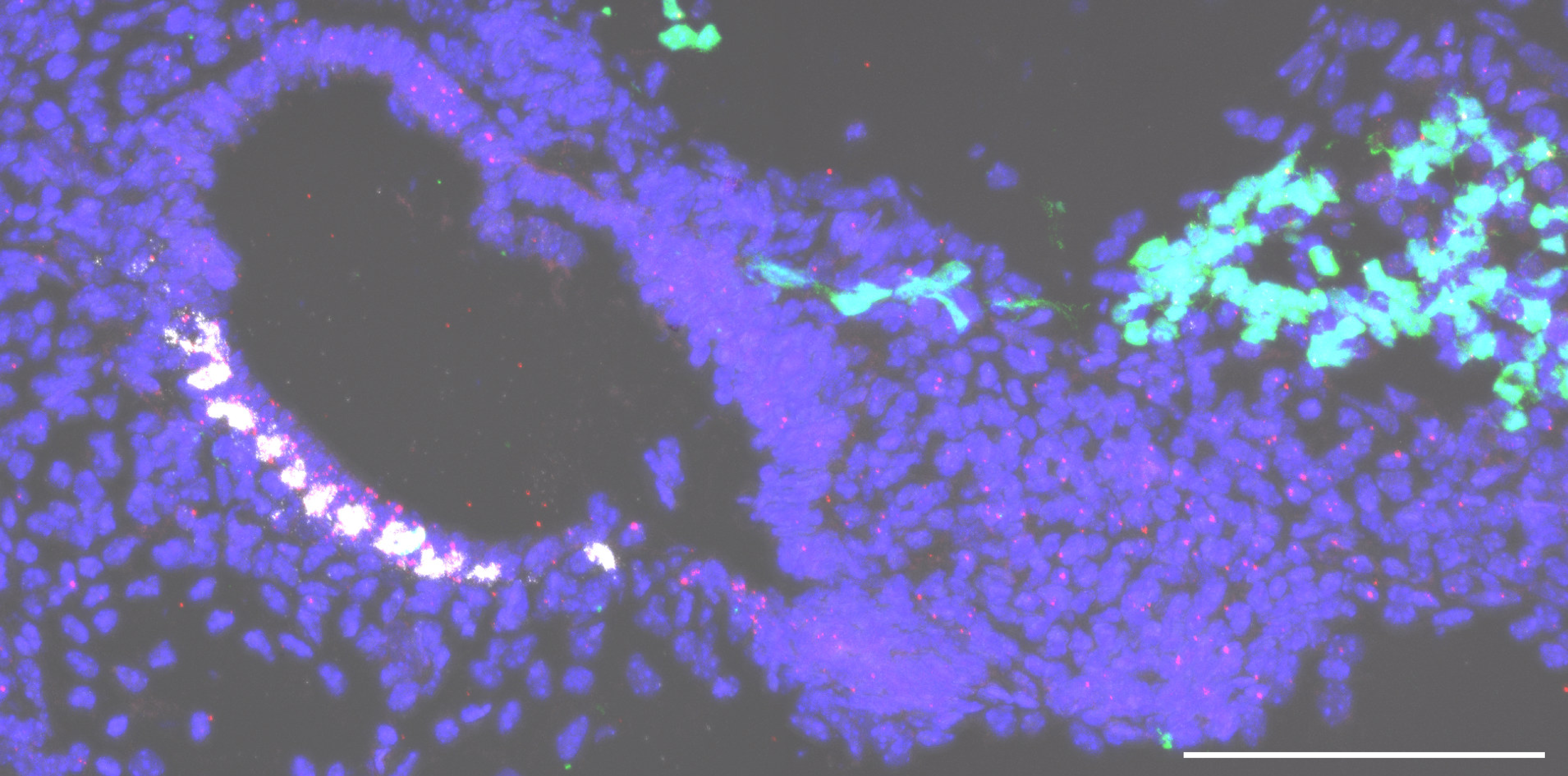

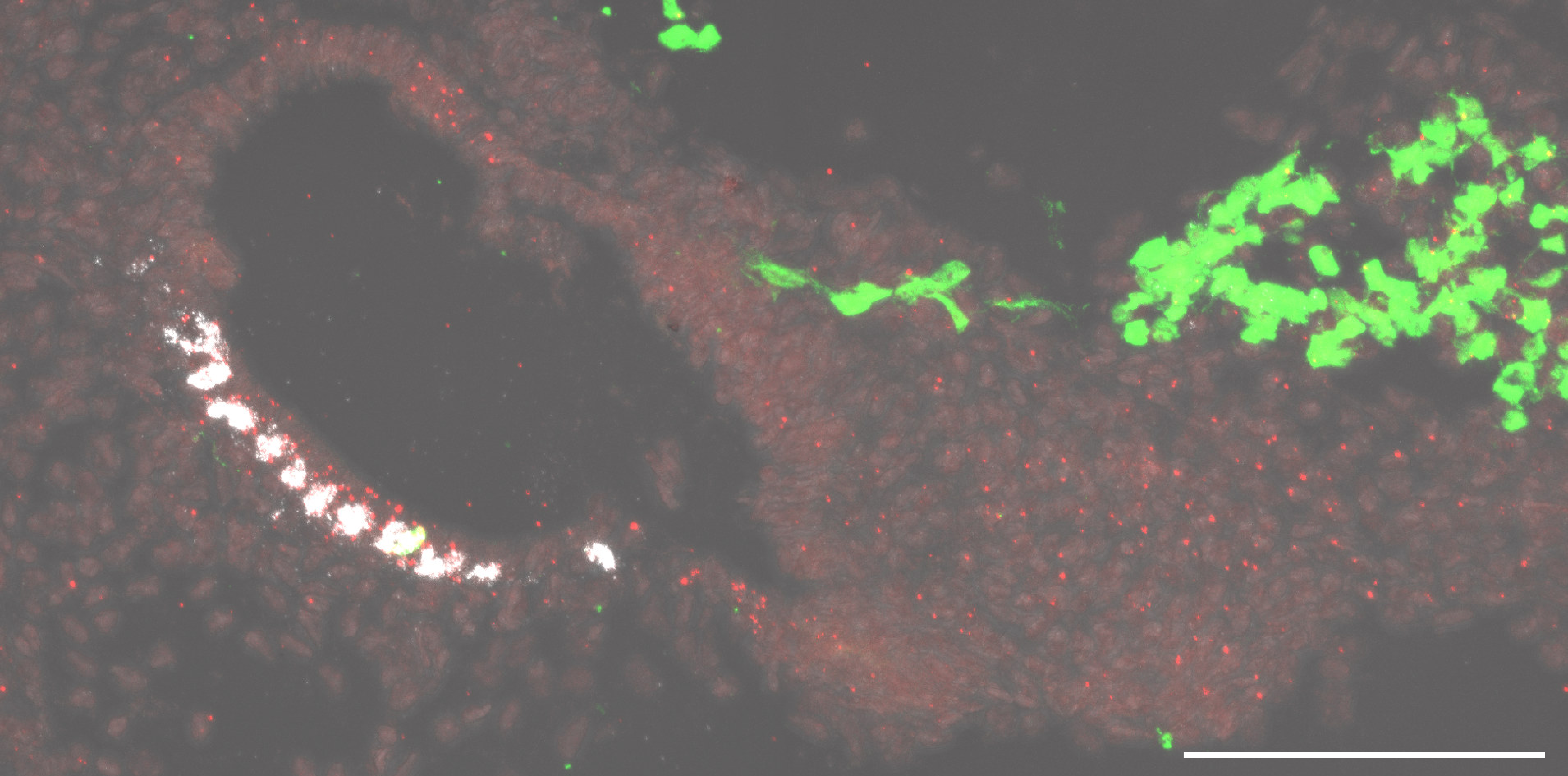

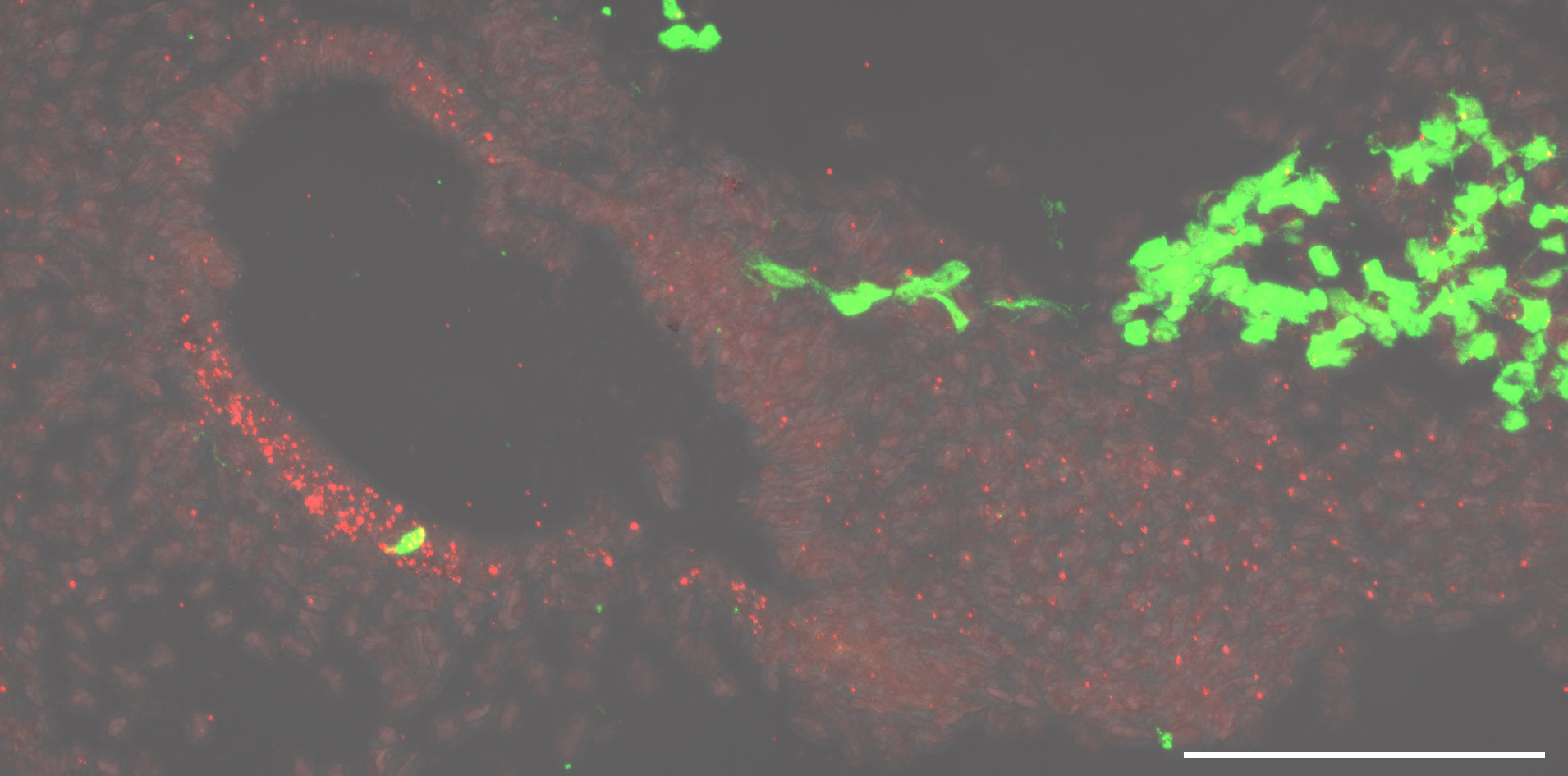

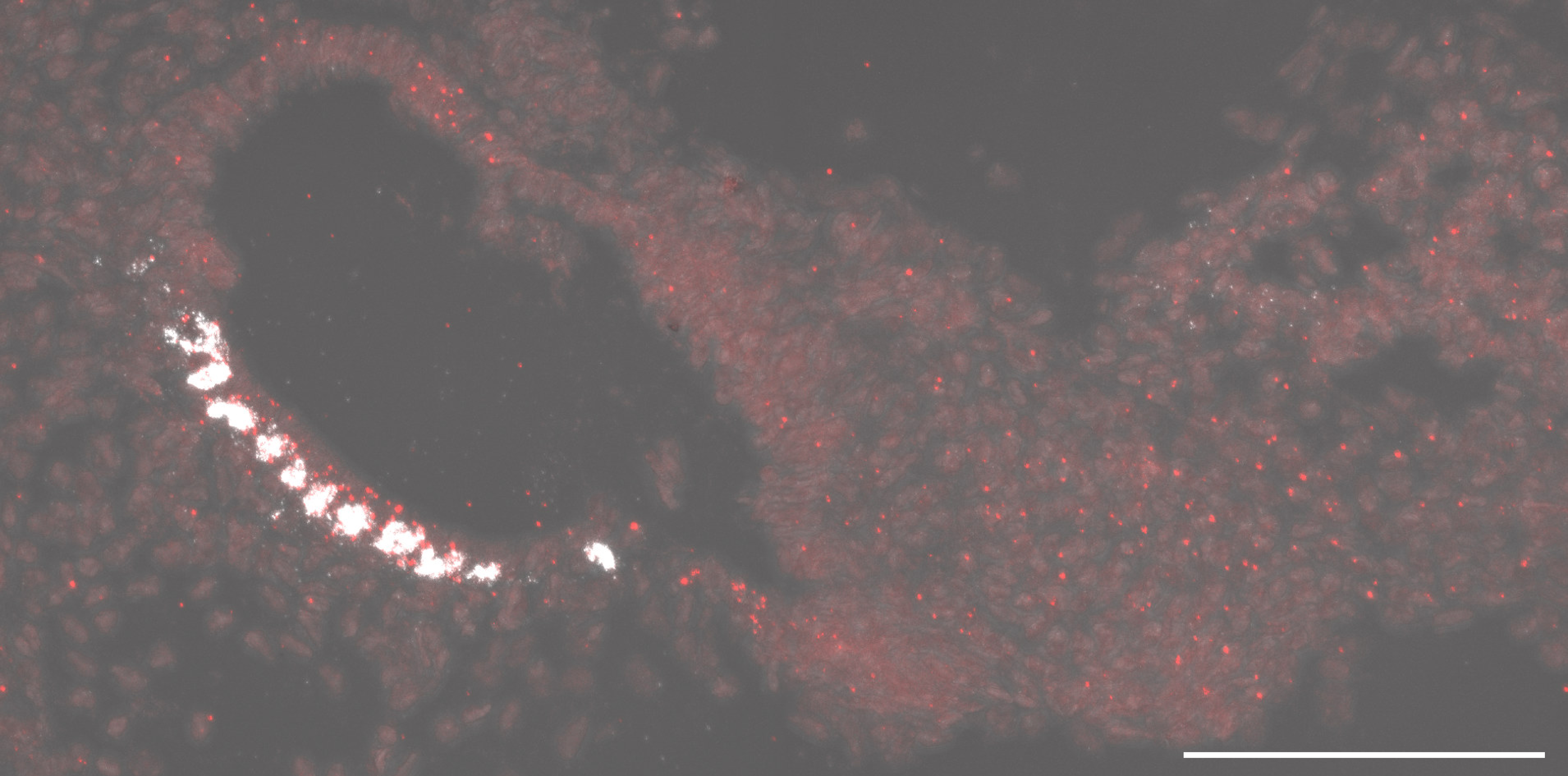

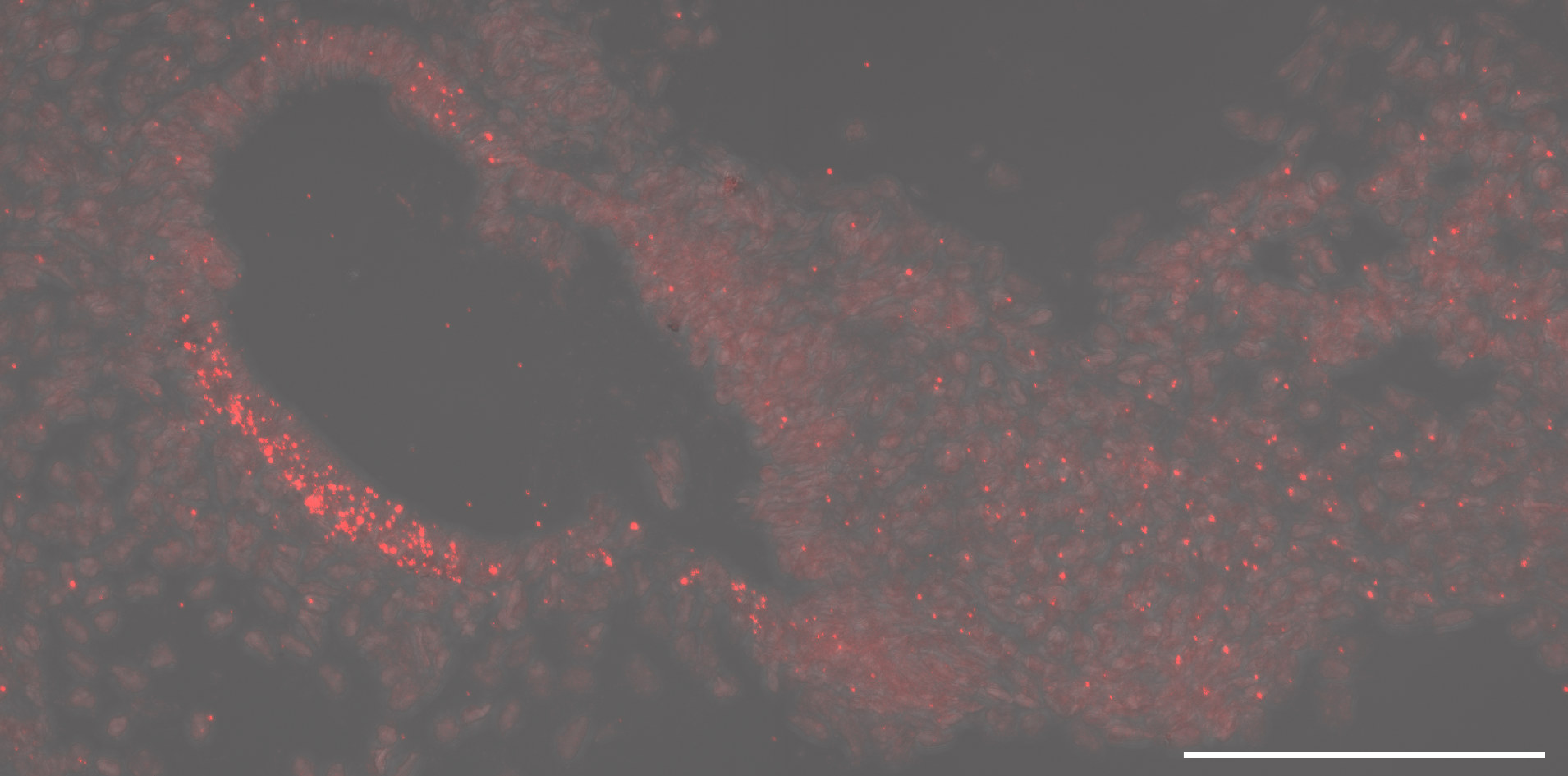

Low-magnification (20x objective) scan of the area between the eye and the optic nerve

- Horizontal section
- T-PMT (transmitted light, brightfield)
- GFP (green, cytoplasmatic, antibody staining)
- SOX10 (red, nuclear staining using antibody)
- *Dct* (white, dotted pattern in the cytoplasm, RNAscope® probe)

Scale bars represent 100 µm

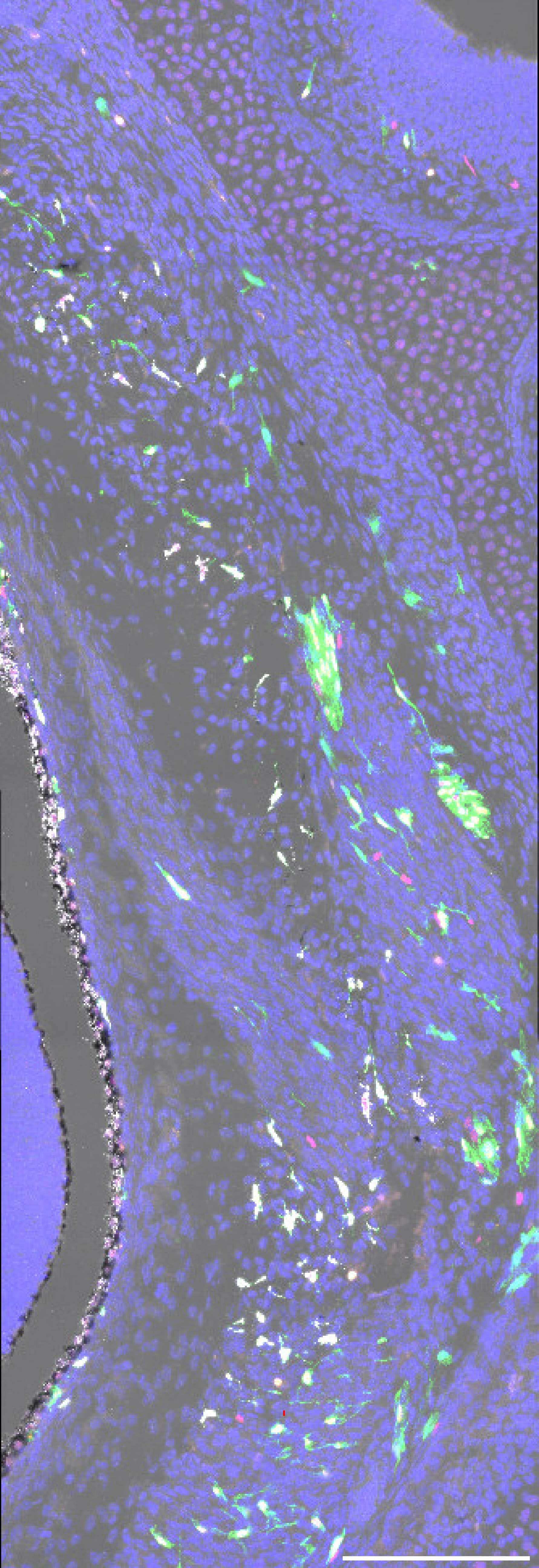

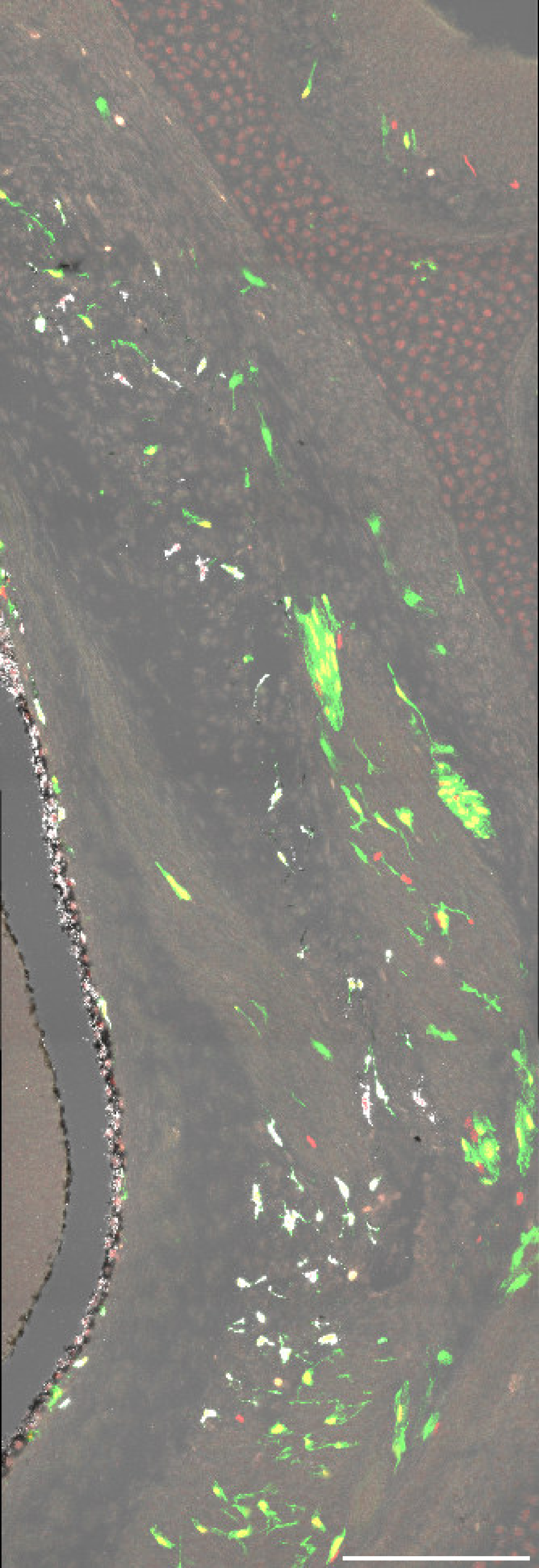

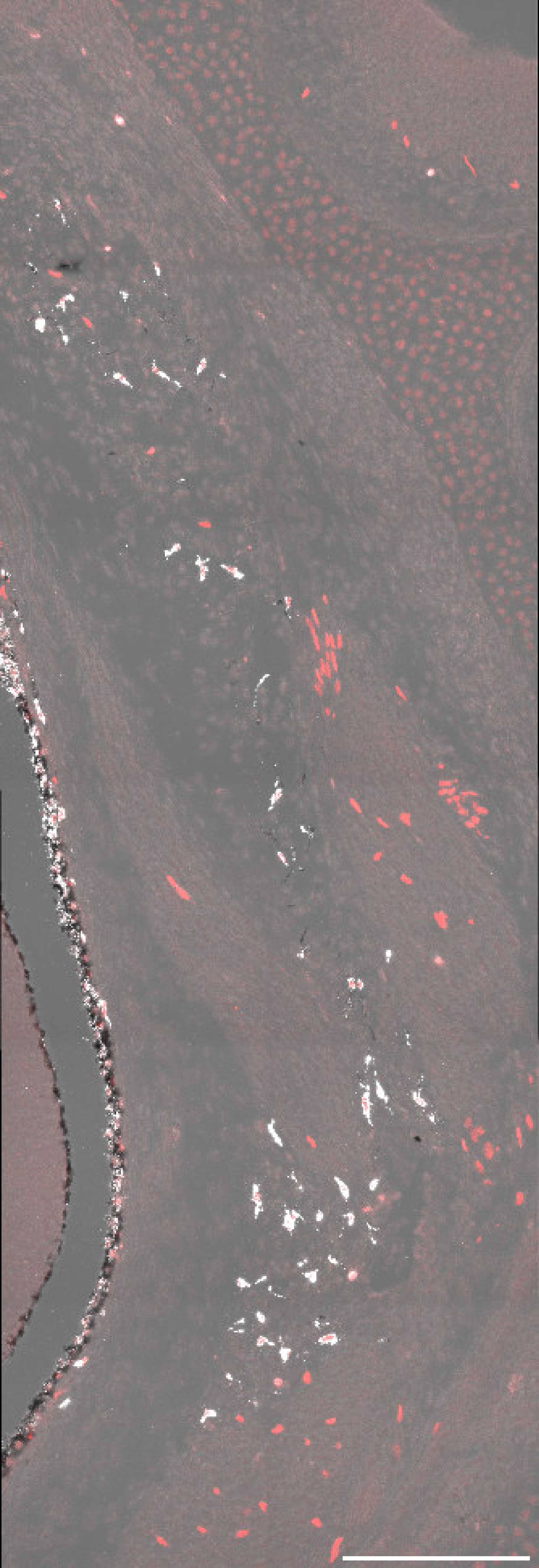

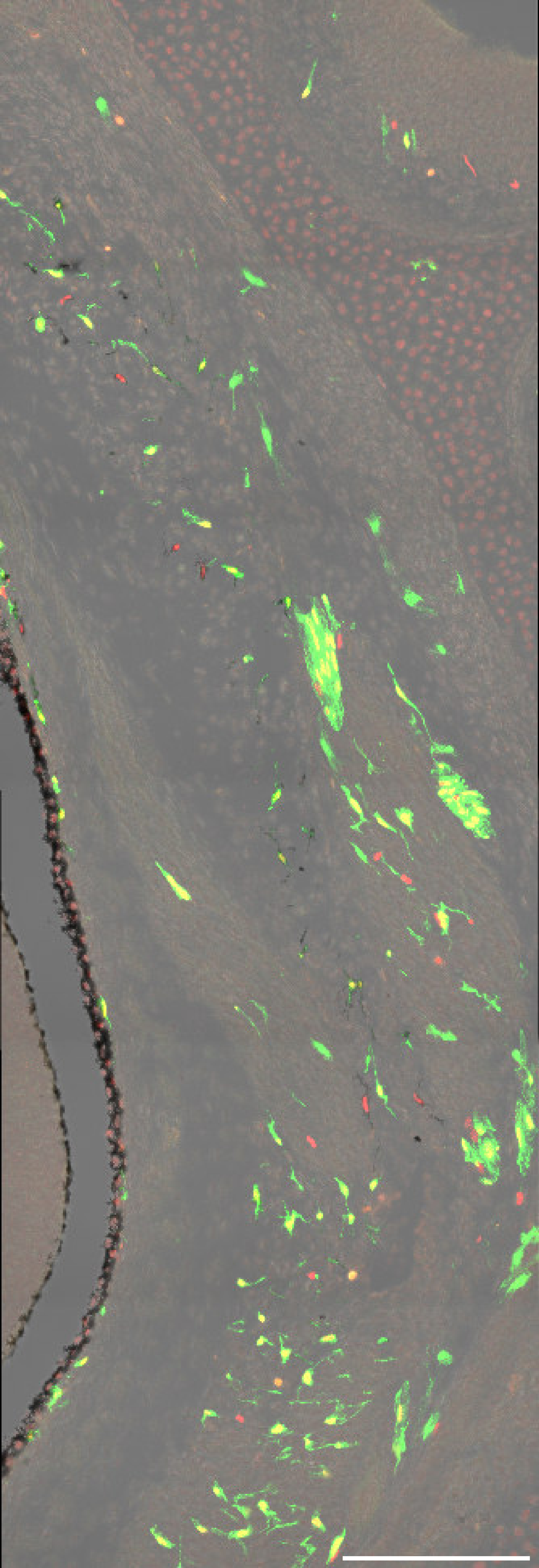

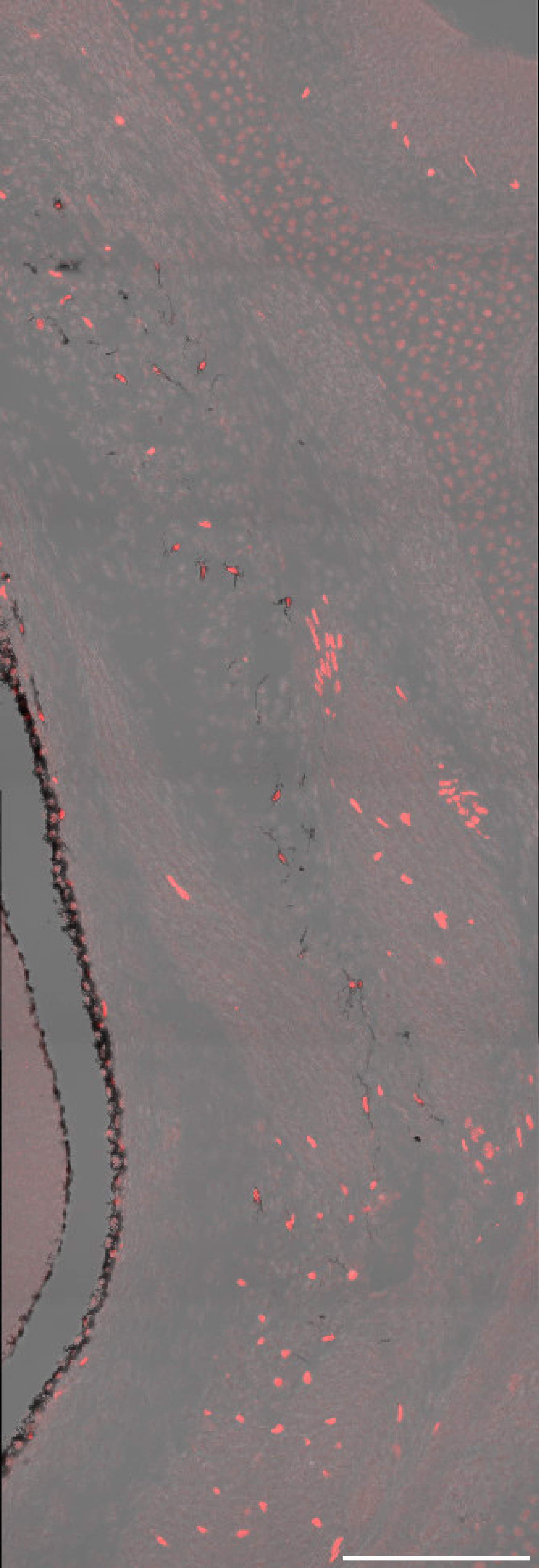

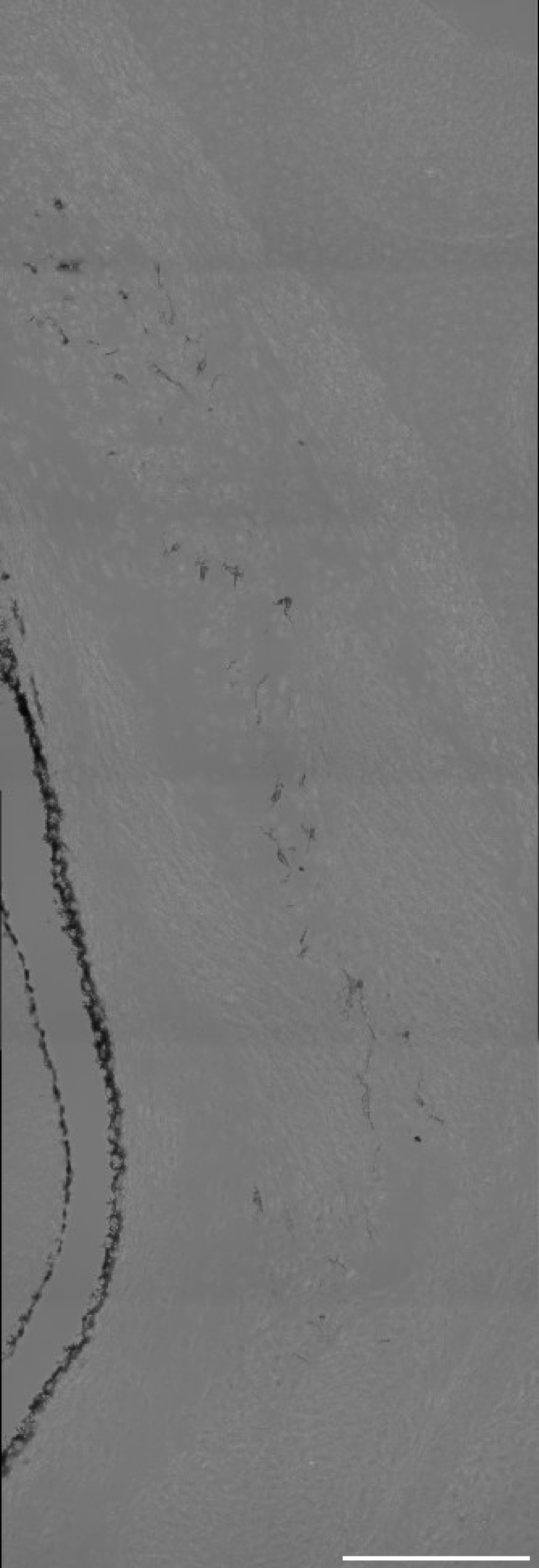

Low-magnification (20x objective) scan of the skin (on the snout)

- Horizontal section
- T-PMT (transmitted light, brightfield)
- GFP (green, cytoplasmatic, antibody staining)
- SOX10 (red, nuclear staining using antibody)
- *Dct* (white, dotted pattern in the cytoplasm, RNA Scope probe)

Scale bars represent 100 μm

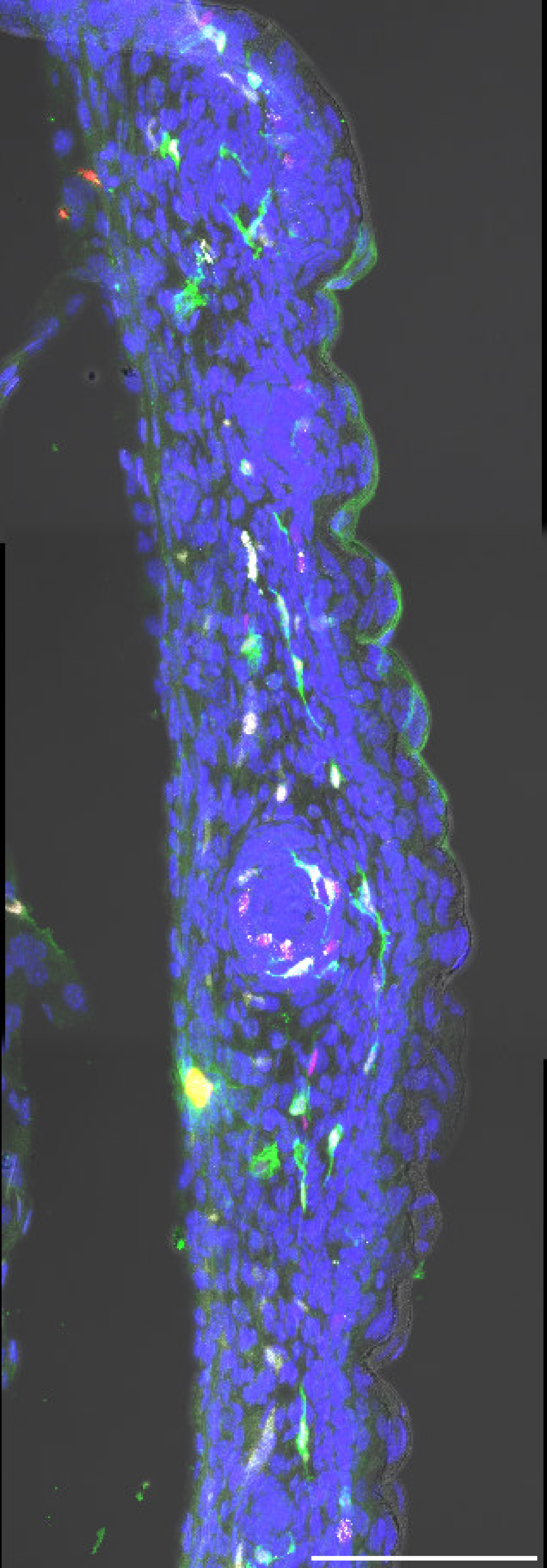

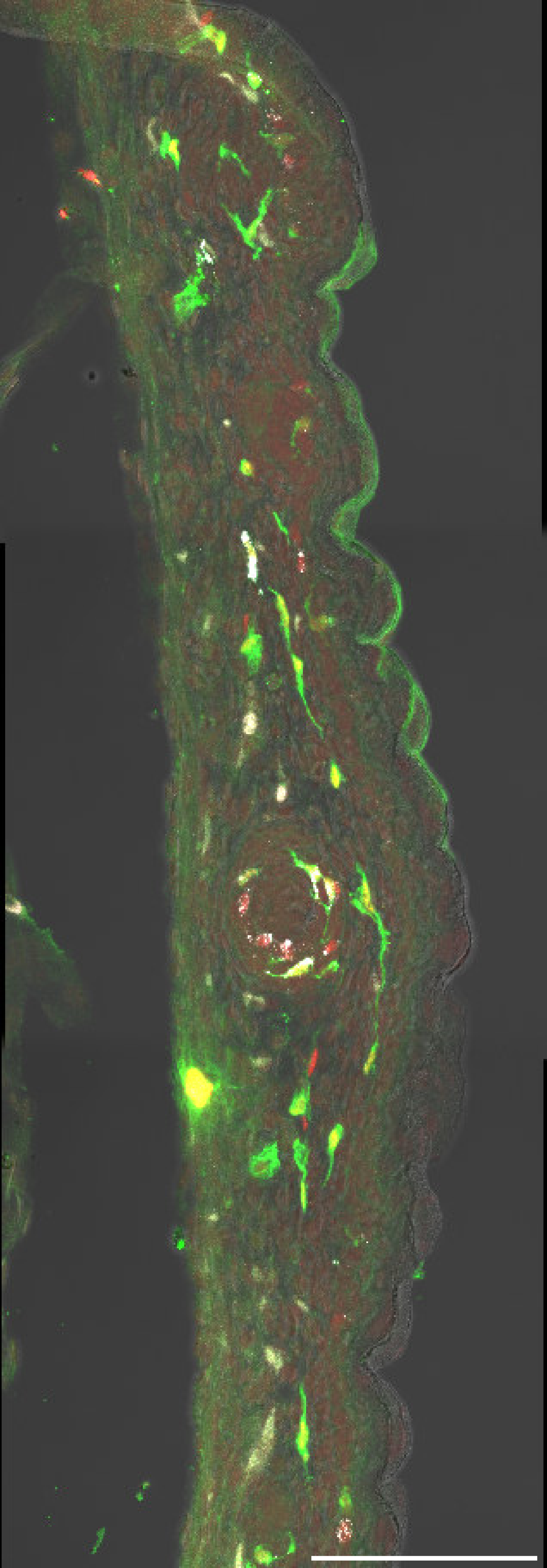

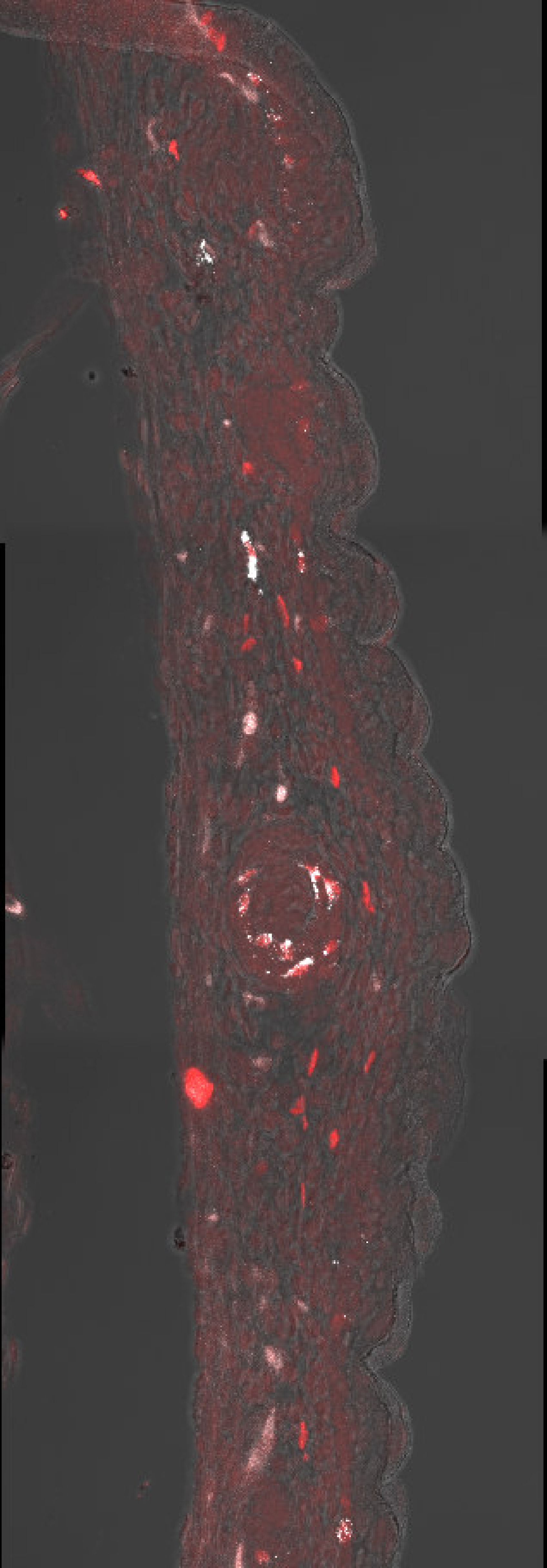

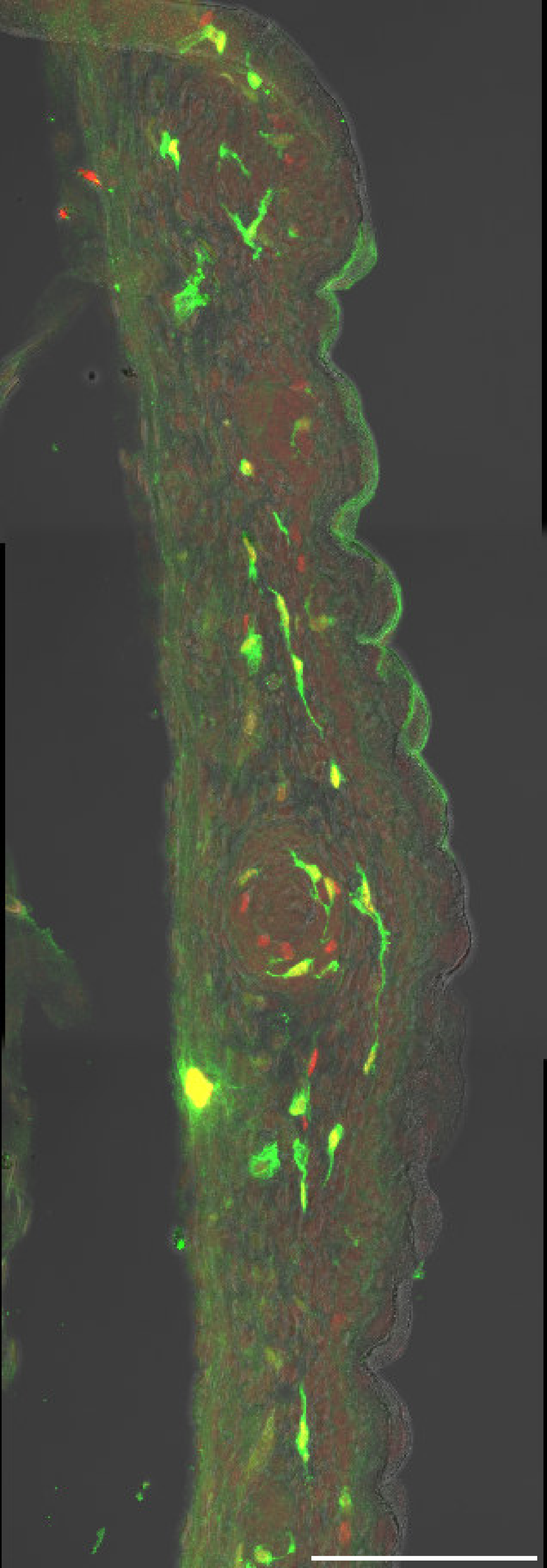

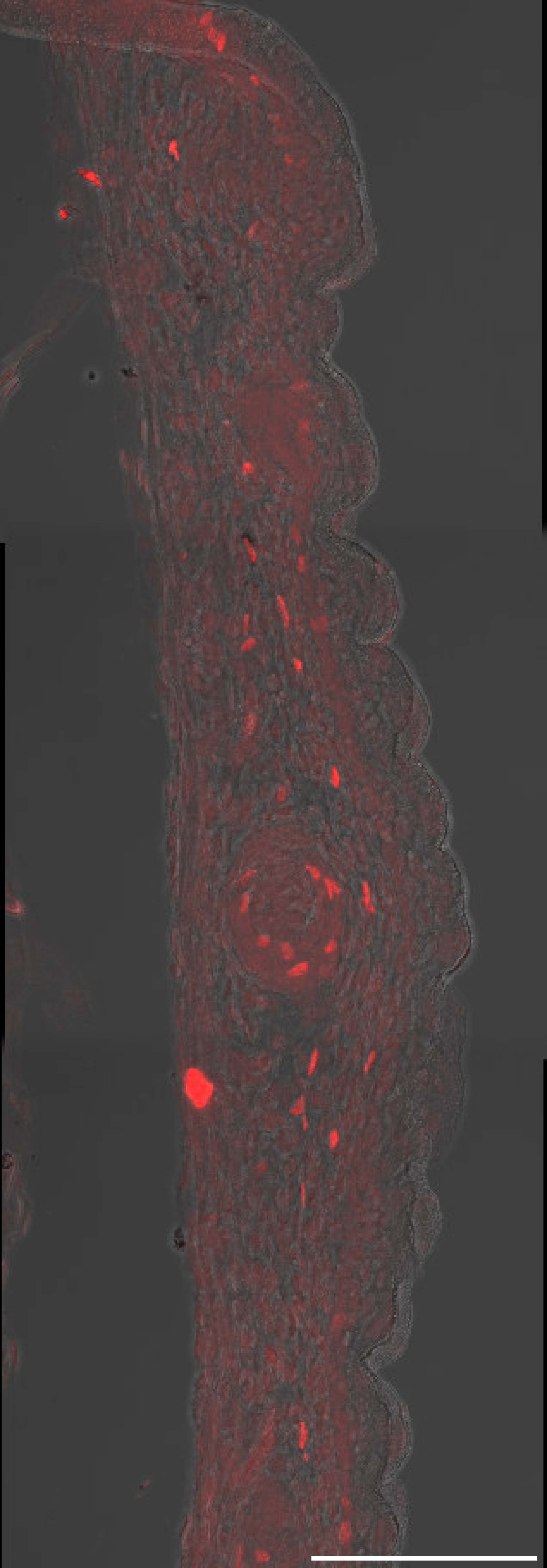

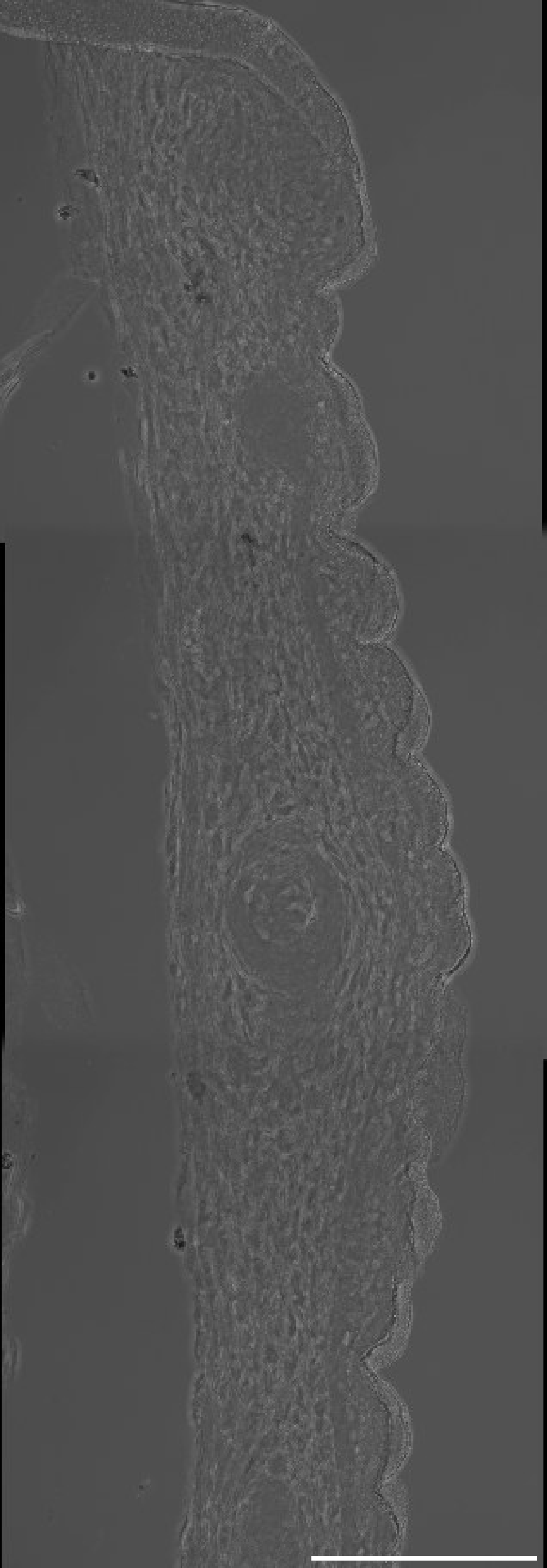

Figure legend:

Supplementary Information File 1: Overview of the embryonic locations containing extracutaneous melanocytes.

Melanocytes were detected using RNAscope® probes (*Dct, Mitf*) and immunohistochemistry (SOX10 and YFP) combined with transmitted light images. *Dct*$^+$ melanocytes were found in the embryonic heart (in the vicinity or associated with the cardiac valves – non-pigmented), meninges of the brain (mainly located along the brain midline – mostly pigmented), inner ear and suborbital locations. Cutaneous melanocytes of the skin showed the same pattern of *Dct*$^+$ signal and SOX10 immunoreactivity as melanocytes found in the extracutaneous locations. Combined RNAscope® for *Dct* and *Mitf* on sections of the embryonic inner ear showed overlap of the two mRNAs, further confirming the use of *Dct* as a melanocytic marker.

Materials and methods:

RNAscope® in situ hybridization

In situ hybridization using the RNAscope® Fluorescent Multiplex Assay kit (version 1) was performed using pretreated cryosections of fixed frozen tissue according to manufacturer's instructions. Probes used are commercially available: Mm-*Mitf*-C2 (Cat No 422501-C2) and Mm-*Dct*-C3 (460461-C3).

Immunofluorescent staining following RNAscope® in situ hybridization

Following the complete hybridization protocol according to manufacturer's instructions, sections were incubated at 4°C, overnight with primary antibodies diluted in 1xPBST (0.1% Tween-20 in 1xPBS). Next, sections were washed in 1xPBST and incubated with secondary antibodies diluted in 1xPBST at room temperature for one hour. Following three washes at room temperature with 1xPBST, DAPI (Sigma, D9542) was applied on the sections at a concentration of 0.5 mg/mL and incubated for 5 min at room temperature. Lastly, the sections were washed three times in 1xPBST and mounted using Mowiöl mounting medium. Primary antibodies used were chicken anti-GFP (Aves Labs Inc. #GFP-1020, 1:500) and goat anti-SOX10 (R&D systems #AF2864, 1:300).For detection of the primary antibodies, secondary antibodies raised in donkey and conjugated with Alexa-488, -555 and -647 fluorophores were used (Molecular Probes, ThermoFisher Scientific, 1:1000).

For detailed description on the tissue preparation, mouse line and ethical permission, please, see the section "Material and Methods" in the manuscript.
